# Plackett-Burman design in the biosynthesis of silver nanoparticles with *Mutisia acuminatta* (Chinchircoma) and preliminary evaluation of its antibacterial activity

**DOI:** 10.12688/f1000research.140883.1

**Published:** 2023-11-13

**Authors:** Luis A. Laime-Oviedo, Carlos A. Arenas-Chávez, Jaime A. Yáñez, Corina A. Vera-Gonzáles

**Affiliations:** 1 Escuela de Ingenieria Quimica ,Facultad de Ingeniería de Procesos, Universidad Nacional de San Agustin de Arequipa, Arequipa, Arequipa, 04000, Peru; 2 Departamento Académico de Biología, Facultad de Ciencias Biológicas, Universidad Nacional de San Agustin de Arequipa, Arequipa, Arequipa, 04000, Peru; 3 Vicerrectorado de Investigación, Universidad Norbert Wiener, Lima, Lima, 15046, Peru; 4 Laboratorio de Preparación, Caracterización e Identificación de Nanomateriales (LAPCINANO), Departamento Academico de Quimica, Facultad de Ciencias Naturales y Formales, Universidad Nacional de San Agustin de Arequipa, Arequipa, Arequipa, 04000, Peru

**Keywords:** Green synthesis, Mutisia acuminatta, silver nanoparticles, Staphylococcus aureus, Escherichia coli

## Abstract

**Background:** The aim of this study was to synthesize silver nanoparticles (AgNPs) using the methanolic fraction of *Mutisia acuminatta* leaves using Plackett-Burman design to optimize process parameters and to evaluate its antibacterial effect.

**Methods:** For the separation of *Mutisia acuminatta* phytoconstituents, chromatographic techniques were used. For characterization and identification, UV - VIS spectrophotometry, FTIR spectrophotometry, Dynamic Light Scattering (DLS) and transmission electron microscopy (TEM) were used. The Plackett-Burman design used polynomial regression statistical analysis to determine the most influential variables.

**Results:** UV-VIS spectroscopy reported an absorbance concerning surface plasmon resonance between 410–420 nm wavelength for the AgNPs. FTIR spectrophotometry reported characteristic peaks in the biosynthesized AgNPs, observing the disappearance of spectral peaks between 1000–1500 cm ^-1^. By UHPLC-MS, caffeic acid derivatives, coumarins, flavonoids, lignans, disaccharide and a complex formed between silver and the solvent (AgCH3CN+) were identified. Using DLS, the AgNPs presented an average hydrodynamic size of 45.91 nm. TEM determined the spherical shape of the AgNPs, presenting diameters in the range of 30 to 60 nm. The biosynthesized AgNPs showed higher antibacterial activity against *Escherichia coli* and *Staphylococcus aureus* than the total extract, the methanolic fraction and pure methanol. The polynomial model in the biosynthesis was validated with an adequate fitting representing the experimental data of the process. The most significant variables for the model obtained were the reaction pH (X _2_) and the concentration of the precursor salt AgNO _3_ (X _6_).

**Conclusions:** The synthesized AgNPs offer a viable option for further development due to the presence of bioactive compounds, adequate characterization and antibacterial activity.

## 1. Introduction

Many researchers have taken an interest in using biological systems such as plants and microorganisms in synthesizing nanoparticles (NPs); there is a growing need to replace chemical synthesis procedures with green synthesis methods for obtaining NPs ^1^^,^^2^ and nanocomposites. ^3^^,^^4^ Green synthesis of NPs is an eco-friendly, non-toxic, fast, and effective method to obtain shape and size-defined metal NPs, acceptable for biomedical applications for their high potential antibacterial effect. ^5^ Since plant extracts contain phytochemicals such as terpenoids, flavonoids, tannins, phenol derivatives, plant enzymes, proteins and reducing sugars, ^6^^–^^8^ these phytochemicals act in reduction as well as protective agents required for synthesis, stabilization of nanoparticles and enhancement of biological properties. ^9^^,^^10^ Flavonoids are compounds synthesized by plants ^8^ and one of the essential bioactive properties is their antioxidant effect ^11^^,^^12^ as well as potential anti-cancer activity. ^13^ On the other hand, in not-so-recent research, the first report was made of the presence of anthocyanins in the genus *Mutisia acuminata* var, in which the chromatographic pattern of flavonoids showed the presence of quercetin, quercetin-3-glucuronide, isorhamnetin-3-glucuronide and pelargonidin diglycoside. The flavonoids were identified by chromatography and spectral data, and agree with the components isolated in other *Mutisia* species. ^14^ Also, a study evaluated the anti-inflammatory effect of *Lepechinia meyenii* (Walp.) by extracting different flavonoid fractions by column and thin layer chromatography. ^15^ Another study determined the presence of functional groups of phenolic compounds and flavonoids that acted as reducing agents in the synthesis of silver nanoparticles (AgNPs) using extracts of *Clinacanthus nutans* leaves and stems. ^2^^,^^16^ Fourier transform infrared (FTIR) spectral recordings identified the functional groups in biomolecules responsible for the precursor salt silver nitrate (AgNO _3_) bioreduction and the protection and stabilization of the synthesized AgNPs. ^17^ The presence of the various peaks determines the possible bioactive compounds that can reduce Ag+ ions and stabilize the formed NPs. ^18^^,^^19^ In the literature, plant compounds are reported to play a vital role in the reduction of metal ions. ^20^^–^^22^

It has also been reported that AgNPs can be synthesized effectively using *Musa paradisiaca* latex peduncle at room temperature; these AgNPs were characterized to be spherical and well-dispersed with an average size of about 40 nm and showed good antimicrobial activity against bacterial species such as *Staphylococcus aureus*, *Pseudomonas aeruginosa*, *Klebsiella* and *Escherichia coli.* ^23^ Another study developed a green method for the synthesis of AgNPs using the aqueous extract of *Laurus nobilis*; these nanoparticles were synthesized with an average size of 19.65 ± 13.49 nm with a spherical shape. The researchers further evaluated how these biomolecules can play an important role in the formation and stabilization of AgNPs. ^24^ Chemical synthesis methods are simple and easy to control; however, they generate toxicity due to unwanted harmful interactions with biological systems. ^25^^–^^27^ On the contrary, the proposal to produce NPs by ecological synthesis using the easy and efficient reduction of metal ions by active molecules from plant extracts results in a cost-effective and environmentally beneficial alternative to the use of chemical and physical methods. ^27^^–^^29^ This will also allow reproducible and controllable synthesis in order to scale up production. ^2^ In this regard, several studies reported the use of various plant species such as *Pelargonium hortorum* leaf, ^30^ green tea, ^31^ *Rosa canina,* ^32^ raspberry extracts, ^32^ the ethanolic extract of *Lepechinia meyenii,* ^33^ and the leaf extract of *Costus igneus* ^33^ as reducing and stabilizing agents. In other studies, silver nanoparticles were obtained with defined spherical shapes and an average size of 13.5 nm and 40 nm, respectively, where the antimicrobial activity on *E. coli* and *S. aureus* strains was evaluated. The results suggest that AgNPs can be used as growth inhibitors of pathogenic microorganisms compared to substances similar to those evaluated. ^2^^,^^10^ In another study, the synthesis of AgNPs using the aqueous extract of *Nepeta deflersiana* was reported, demonstrating the induction of cell death of human cervical cancer (HeLA) cells after treatment with the NPs. ^34^

Recent studies explain the management of physical and chemical parameters for the use of optimal conditions in the preparation of the extract of a plant sample and the synthesis of AgNPs, making use of statistical methodologies, where factorial designs and polynomial regression statistical techniques were employed to optimize the biosynthesis parameters of AgNPs. ^2^ Therefore, some studies mention the use of scaling and optimization tools and the need for the use of experimental methods and designs in bioprocesses: complete factorial design, fractional factorial design, Plackett-Burman design (PBD), Taguchi design, Box-Behnken design (BB) and Central Composite design (CCD); furthermore these lead to obtaining a polynomial equation representative of the experimental data to describe the behavior of the parameters, and the adequacy of the model is obtained by applying an analysis of variance. ^17^^,^^35^ In another work, the reaction variables that were most influential on the yield of AgNPs were determined to be the concentration of aqueous silver nitrate (AgNO _3_) solution, the concentration of plant extract, and reaction time. ^33^ On the other hand, a study was reported where the ultrasound-assisted extraction of olive leaf flavonoids was optimized by response surface methodology, and the flavonoid compounds, and their antioxidant and anticancer activities were investigated by high-performance liquid chromatography. ^36^ Another study used gallic acid (GA), where many NPs with a smaller average size were obtained at the GA concentration of 7 mM and Zeta potential values indicated that AgNPs had good stability under all conditions. ^37^

Furthermore, green chemistry methods have been developed for the biosynthesis of AgNPs using aqueous extracts from various plant species. These nanoparticles synthesized with biological reducing agents have shown considerable average sizes, for example, a satisfactory average size of 19.65 ± 13.49 nm with defined spherical shapes was reported. ^24^ In another study, AgNPs were effectively synthesized using *Musa paradisiaca* latex peduncle at room temperature with spherical shape and were dispersed nicely with an average size of about 40 nm showing good antimicrobial activity towards bacterial species such as *Staphylococcus aureus*, *Pseudomonas aeruginosa*, *Klebsiella* and *E. coli.* ^23^ Experimental designs such as the Plackett and Burman design (PBD) have been used to study the significance of variables in the biosynthesis of AgNPs. PBD develops experiments by minimizing the number of trials, allowing a multifactorial analysis of the variables analyzed for the segregation and evaluation of their significance. ^8^ The parameters and significant effects of the variables in the biosynthesis of AgNPs with the methanolic fraction of *Mutisia acuminatta* leaves were determined using the PBD factorial design. ^38^ The aim of this study is to optimize the AgNPs biosynthesis process using a polynomial regression model that is representative of the experimental data using *Mutisia acuminatta* leaves as well as to evaluate the antibacterial activity against strains of *E. coli* and *S. aureus.*

## 2. Methods

### 2.1 Preparation of the total extract and obtaining the methanol fraction (MF)

The leaves of the Peruvian medicinal plant *Mutisia acuminatta* (chinchircoma) were washed, sorted, dried, pulverized, and sieved through a Tyler #100 mesh, yielding 60.00 g of powdered plant material. The powder was degreased with 300 mL of petroleum ether, shaken until homogenization, and subjected to ultrasound treatment in a bath (Branson model CPX5800H) under the following conditions: 25°C, 10 min, 40 kHz, and moderate power. The degreased sample was then filtered using Whatman fast passage paper and dried at room temperature. To prepare the extract, 58.1734 g of the dried defatted sample was added to 290 mL of 80% methanol and sonicated. The extract was centrifuged at 5000 RPM for 5 min in a swing-rotor centrifuge (Eppendorf), and the supernatant was collected for further fractionation by chromatography. Gradient column chromatography (CC) was performed using the following solvents as the mobile phase: petroleum ether, ethyl acetate, and methanol, in order of increasing polarity. The fractions obtained were monitored by thin-layer chromatography (TLC), and the fractions with the highest polarity were selected.

### 2.2 Identification of flavonoids by UHPLC - MS

To prepare the samples, 100 μL of each solution was taken and diluted with methanol in a ratio of 1:10. The resulting mixture was then sonicated for 5 minutes and filtered through a 0.25 μm PVDF disc filter into an HPLC vial. The samples were analyzed using a UHPLC chromatograph model Dionex Ultimate 3000 UHPLC system (Thermo Scientific), equipped with a Luna Omega C18 100 Å, Phenomenex column (150 x 2.1 mm, 1.6 μm) kept at 40 °C, and an injection volume of 2 μL. A gradient elution system was used with eluent A (1% HCOOH in H2O) and eluent B (1% HCOOH in ACN) at a flow rate of 0.2 mL/min. The coupled mass spectrometer model Q Exactive Plus (Thermo Scientific) was used to detect the compounds. Full MS scan parameters included a range of 130-1600 m/z, a resolution of 70,000, micro scans of 1, an automatic gain control (AGC target) of 1 × 10 ^6^, and a maximum IT of 100 ms.

### 2.3 Biosynthesis of silver nanoparticles

The biosynthesis of silver nanoparticles was carried out using different volumes (100-150 μL) of the FM methanolic fraction solution, which were added to the AgNO _3_ precursor salt (1 mM), under the same synthesis conditions as described earlier. ^1^ Each experimental trial was developed according to the Plackett and Burman factorial design. For biosynthesis, 15 mL of AgNO3 salt (1 mM) was used as a precursor, and 5 mL of 20% EMT from the plant species ( *Mutisia acuminatta*) was added. This solution was previously alkalinized by adding drops of NaOH (0.1 N) until a basic pH of 8-9 was obtained, using a multiparameter (Thermo Scientific model Star PRO “Orion”). The other synthesis conditions were as follows: temperature 45-48°C, agitation speed 600 rpm, and a synthesis time of 90 min. Agitation was carried out using a magnetic plate with a temperature sensor (Thermo Scientific model “Super Nuova +”). All solutions and dilutions were prepared using deionized water with a conductivity of 0.050 μS, supplied by the water purification system (Thermo Scientific, model “Smart 2 Pure”).

### 2.4 Characterization of biosynthetized AgNPs

**2.4.1 Ultraviolet-Visible Spectrophotometry (UV-VIS)**

The UV-VIS spectrophotometer (Thermo Scientific brand, model Evolution 220) was used to read and monitor the biosynthesized AgNPs. Each reading was taken from a 1000 uL sample, which was diluted with an 80% methanolic solution in a 1:2 ratio for each sample analyzed. Spectral scanning was performed between 350 nm - 750 nm to determine the maximum peak absorbance as a function of wavelength, corresponding to the surface plasmon resonance (SPR) of the AgNPs, in accordance with the Plackett and Burman design (PBD). The spectrophotometric readings were taken using a quartz cuvette with a step width of 1 cm. The maximum absorbance was determined at a wavelength of 411 nm, and the target used for each spectral sweep was an 80% methanolic solution. Thermo INSIGHT software was used for spectral processing.

**2.4.2 Fourier transform infrared spectrophotometry (FT-IR)**

The FT-IR spectrophotometric analysis was performed using a Thermo Scientific Nicole iS50 Analytical model equipped with “OMNIC Spectra” software for data analysis. Aliquots of the 20% ETM, FM, and AgNPs samples were individually placed on the diamond ATR lens for each reading. Prior to each measurement, a spectral background (BG) was recorded in transmittance (T) mode within the mid-IR range (4000 cm ^-1^ to 400 cm ^-1^). The diamond ATR accessory was cleaned with Kimtech absorbent cloths and 98% absolute ethyl alcohol to prevent cross-contamination between samples. A final BG spectral background was taken after each data collection.

**2.4.3 Dynamic light scattering (DLS)**

The hydrodynamic size of the biosynthesized AgNPs was measured using dynamic light scattering (DLS) with a Malvern Zetasizer Nano ZSP instrument. Prior to analysis, the samples were filtered using a 10 mL syringe and a 0.22 μm pore size filter. For each measurement, 1.5 mL of the filtered sample was added to a polypropylene cuvette. The instrument software required several input parameters for the analysis, including the refractive index (IR) of silver (1.330), the dispersant medium (methanol), and the dynamic viscosity (Cp) of the dispersant medium (0.587 for 20°C). The software generated a histogram plot of the size distribution, which showed the percentage of scattered light intensity (%) as a function of the hydrodynamic particle diameter distribution (d.nm).

**2.4.4 Transmission electron microscopy (TEM)**

The biosynthesized NPsAg were analyzed using an atomic resolution TEM (Thermo Scientific, model Talos F200i) with a working voltage of 20 to 200 kV and a line resolution of less than 0.10 nm. For analysis, the sample was supported on a MESH 300 CU Grid (TEM). Prior to analysis, the AgNPs sample was centrifuged at 5000 RPM using a tilting angle rotor centrifuge, and the solid phase obtained (precipitate) was separated from the liquid phase. This solid phase was then washed with pure methanol three times, and a 10 uL sample was taken and supported on the MESH 300 CU Grid. The sample was dried for 48 hours in a chamber with a relative humidity (RH) of 30% and a temperature of 25°C. To ensure that the AgNPs were not conglomerated or in a state of structural aggregation at the bond level, an ultrasound pretreatment (BRANSON 5800) was performed for approximately 15 minutes.

### 2.5 Experimental design

**2.5.1 Software**

Minitab 19 software was used for assay development, Plackett-Burman experimental design (PBD), and analysis of variance (ANOVA). The software was also used to generate 3D response surface models and 2D contour plots, perform correlation analysis of variables, and visualize the interaction of results.

**2.5.2 Plackett - Burman design (PBD)**

The Plackett-Burman experimental design (PBD) ^38^ was used to investigate the influence of six factors on the absorbance (411nm) as the dependent variable for the biosynthesis of silver nanoparticles. The six factors included were stirring speed (X1, RPM), potential hydrogen (X2, pH), synthesis temperature (X3, °C), synthesis time (X4, min), volume of methanolic fraction (X5, μL), and concentration of AgNO _3_ (X6, mM). Twelve factorial tests and three tests with PBD center points were conducted to determine the most significant factors, as presented in Table 1 and Table 2 of the experimental trials. The polynomial regression model shown in equation 1 was used to analyze the data:

$$\hat{Y}=b+b\_1\;X\_1+b\_2\;X\_2+\cdots +b\_k\;X\_k+Z\_r$$ (1)

**Table 1.** Levels of variables evaluated by the Plackett-Burman design (PBD) for the biosynthesis of AgNPs.

Name of the factors			Levels		
N°	Independent variables	Unit	-1	0	+1
1	X1: Agitation Speed	RPM	400	550	700
2	X2: Hydrogen Potential	pH	8	8.5	9
3	X3: Synthesis Temperature	°C	45	50	55
4	X4: Synthesis Time	Min	30	60	90
5	X5: Volume of Methanolic Fraction	uL	150	175	200
6	X6: AgNO _3_ Concentration	mM	0.5	1.0	1.5

N°	Dependent variables	Unit	Restriction		
1	Y: Absorbance (411 nm)	U.A	ABS 411 nm		

**Table 2.** Experimental trials of the Plackett-Burman factorial design (PBD).

Essay	Plackett-Burman design variables							
N°	X _0_	X _1_	X _2_	X _3_	X _4_	X _5_	X _6_	Y (Abs 411 nm)
1	1	+1	-1	+1	-1	-1	-1	0.0604
2	1	+1	+1	-1	+1	-1	-1	0.1547
3	1	-1	+1	+1	-1	+1	-1	0.9964
4	1	+1	-1	+1	+1	-1	+1	0.1881
5	1	+1	+1	-1	+1	+1	-1	0.8948
6	1	+1	+1	+1	-1	+1	+1	1.2908
7	1	-1	+1	+1	+1	-1	+1	1.5373
8	1	-1	-1	+1	+1	+1	-1	0.1829
9	1	-1	-1	-1	+1	+1	+1	0.3085
10	1	+1	-1	-1	-1	+1	+1	0.6576
11	1	-1	+1	-1	-1	-1	+1	0.8053
12	1	-1	-1	-1	-1	-1	-1	0.0235
13	1	0	0	0	0	0	0	0.5341
14	1	0	0	0	0	0	0	0.4032
15	1	0	0	0	0	0	0	0.5375
Σ (Sum)	8	0	0	0	0	0	0	

In addition, the effects of each variable were determined, including the degrees of freedom (df), the Sum of Squares (SC), the Mean of the Sum of Squares (MC), and the calculation of the Fo statistic. A Fisher’s test was also conducted using the F statistic according to tables at the 95% significance level (p-value < 0.05) with 1 and 2 degrees of freedom, respectively. The F statistic was used to evaluate the significance of the biosynthesis variables, which were considered significant for Fo > F (tables). The statistical software Minitab 19 Ink was used for assay development, PBD, ANOVA, analysis of 3D response surface models and 2D contour plots, correlation analysis of variables, and graphical interaction of results.

**2.5.3 Antibacterial evaluation**

**2.5.3.1 Zone of inhibition method and sensitivity analysis**

Certified strains of *Staphylococcus aureu*s ATCC ^®^ 25923 (Epower) and *Escherichia coli* ATCC 33876 were obtained from Sigma Aldrich. All glassware was sterilized in a unidirectional laminar flow chamber at 80°C for 15 min in a Pasteur oven prior to use. The culture medium (Mueller Hinton) was prepared by dissolving 34 g per 1000 mL of distilled water under agitation and constant boiling, following the manufacturer’s specifications (Merck KGaA). The medium was then sterilized using an LA autoclave (Biogenics brand, model AVDA 20-D) at 121°C for 15 min and 15 lb of pressure. Inoculum preparation followed the laboratory’s recommendations for acquiring *Escherichia coli* and *Staphylococcus aureus* strains, with the lyophilized strains reactivated in previously sterile 0.1% peptone water. To do this, 0.1 g of peptone (Liofilchem brand) was weighed and diluted in 100 mL of double distilled water and used as a diluent for the lyophilized strains. Sterile swabs were moistened with the inoculum, distributed and sown in test tubes with slant agar, covered, and incubated at 37°C. Approximately 15 mL of the Mueller Hinton agar medium was then poured into each sterile petri dish and allowed to cool before use.

## 3. Results

### 3.1 Preparation of the total methanolic extract (EMT) and obtaining the methanolic fraction (FM)

**Figure 1.**
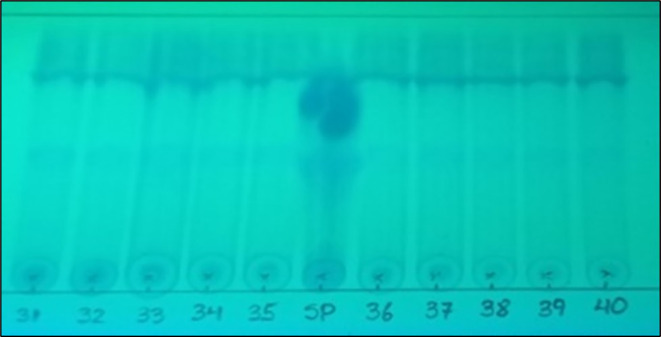
TLC (thin layer chromatography) plate of the methanolic fraction.

The defatted plant sample showed a very intense dark green color after drying at room temperature, and its dry weight was recorded using an analytical balance, yielding a total of 58.1730 g, which was used to prepare a 20% total methanolic extract (TME). The TME obtained had an intense dark brownish-green color. The yield of the crude extract from the methanolic extraction was 70%, which was prepared using ultrasound under the following conditions: 35°C, 45 min, 40 kHz, and moderate power. The FM was then obtained in a series of test tubes using column chromatography (LC) eluates with varying concentrations and polarities of solvents used as the mobile phase. The eluates obtained were grouped based on their characteristics observed in the TLC plates, which showed a variable intensity of coloration ranging from brown to light yellow. The chromatographic plates (TLC) were developed up to 85% of the total height in a horizontal glass chamber (10 cm × 15 cm) with the medium saturated with the mobile phase for 30 min at room temperature and 60% relative humidity. The TLC plates were then dried with airflow and developed with UV light. Figure 1 shows the TLC plate of tubes 31-40 corresponding to the methanolic fraction.

### 3.2 Metabolite identification by High-Performance Liquid Chromatography coupled to mass spectrophotometry (HPLC - MS)

**Figure 2.**
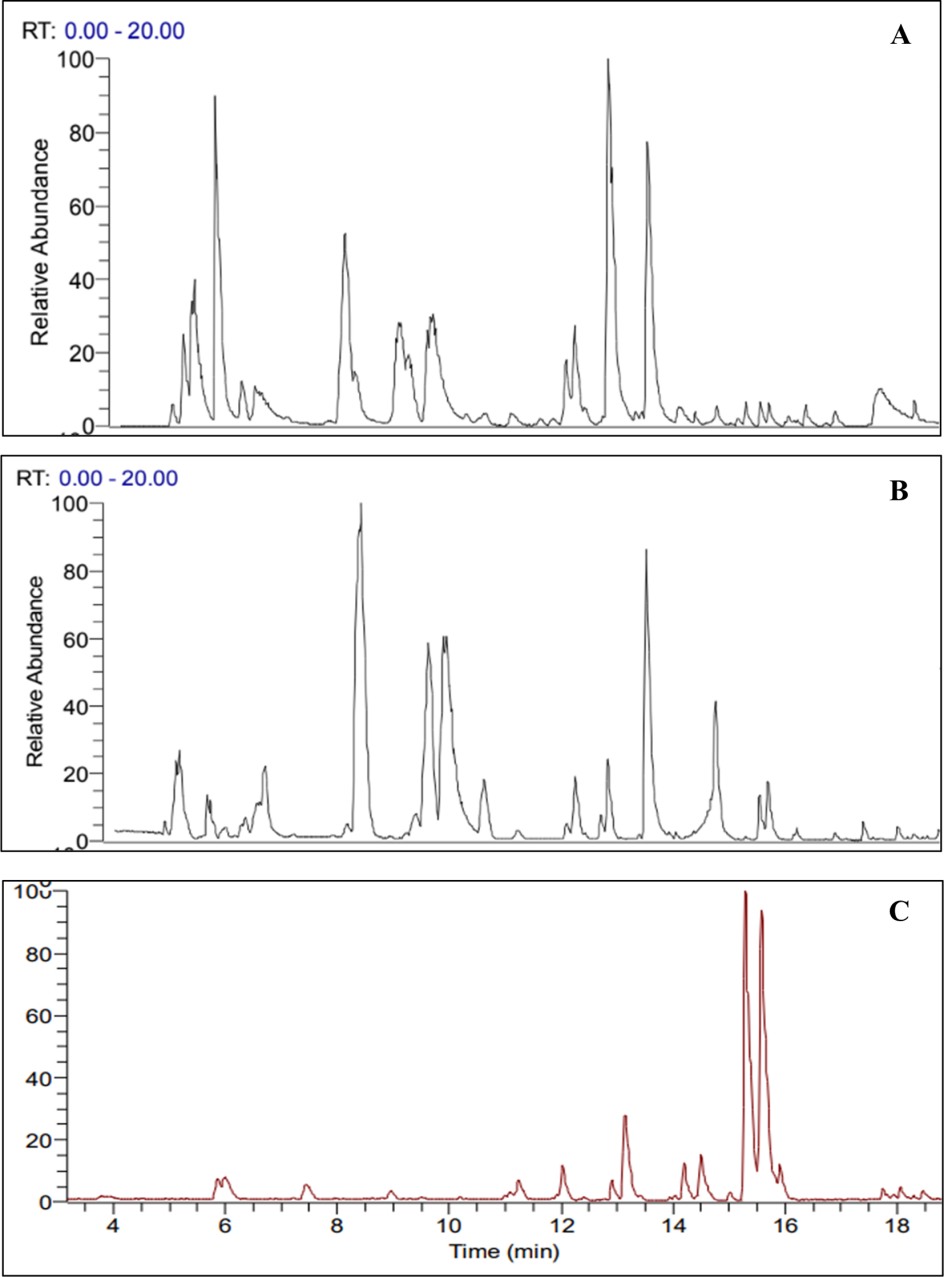
A) Chromatogram of the total methanolic extract at 20% (EMT 20%) of *Mutisia acuminata.* B) Chromatogram of the methanolic fraction (FM) of *Mutisia acuminata.* and C) Chromatogram of the biosynthesized silver nanoparticles (AgNPs-FM) of *Mutisia acuminata.*

Figure 2A, 2B, and 2C show the total ionic current chromatograms (TIC) of samples obtained from *Mutisia acuminata* leaves, including the total methanolic extract at 20% (EMT), the methanolic fraction (FM), and the biosynthesized silver nanoparticles (AgNPs - FM). ^73^ The number of signals and their intensity in the chromatograms varied according to the concentration and structural characteristics of the molecules present in the samples. The total methanolic extract ( Figure 2A) showed a higher number of signals in the chromatogram, detecting derivatives of caffeic acid, coumarins, flavonoids, and lignans in the zone between 0-15 minutes. After 15 minutes, many unknown compounds were detected, corresponding to new nitrogenous derivatives of coumarins. The FM ( Figure 2B) exhibited a similar profile to the EMT, where several of the compounds detected in the EMT were not observed, especially in the zone above 15 minutes. Finally, in Figure 2C (AgNPs - FM), the number of detected signals was very low, detecting a disaccharide, coumarins, and a complex formed between silver and the solvent AgCH _3_CN+ at t = 1.95 min. The isomeric compounds at m/z 430 (t = 15.27 min and t = 15.56 min) were the most intense signals in this sample, whose structures could not be identified. There is a high possibility that these are new coumarin-derived compounds.

### 3.3 UV-VIS Spectrophotometry

**Figure 3.**
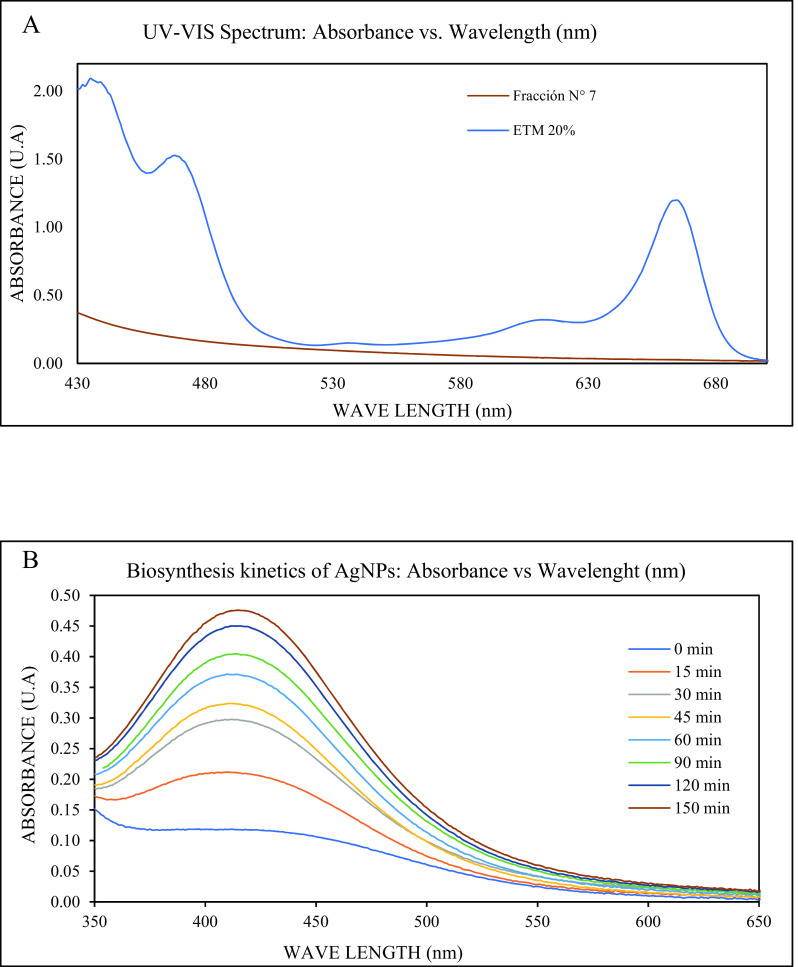
A) UV-VIS spectra of the 20% total methanolic extract (TME) of *Mutisia acumintta* and B) UV - VIS spectra of the biosynthesized AgNPs showing the absorbance at different time intervals.

Figure 3A displays the UV-Visible spectral scan of the 20% EMT of *Mutisia acuminata* obtained from 400 nm to 750 nm wavelength range. The main absorption peak with a maximum absorbance of around 650-680 nm and a secondary peak at around 470 nm corresponds to the phytochemical components of the TME, including chlorophyll. These specific UV-VIS absorbance peaks are directly related to the time, extraction method, and concentration of the methanolic extract obtained. Additionally, in Figure 3A, the UV-VIS spectrum of the FM obtained by LC is shown, where no peak can be observed between 400-420 nm, corresponding to the RPS of the AgNPs. This finding confirms that the starting EMT and the FM obtained do not contain the characteristic peak of the silver nanoparticle.

### 3.4 FT-IR Spectroscopy

**Figure 4.**
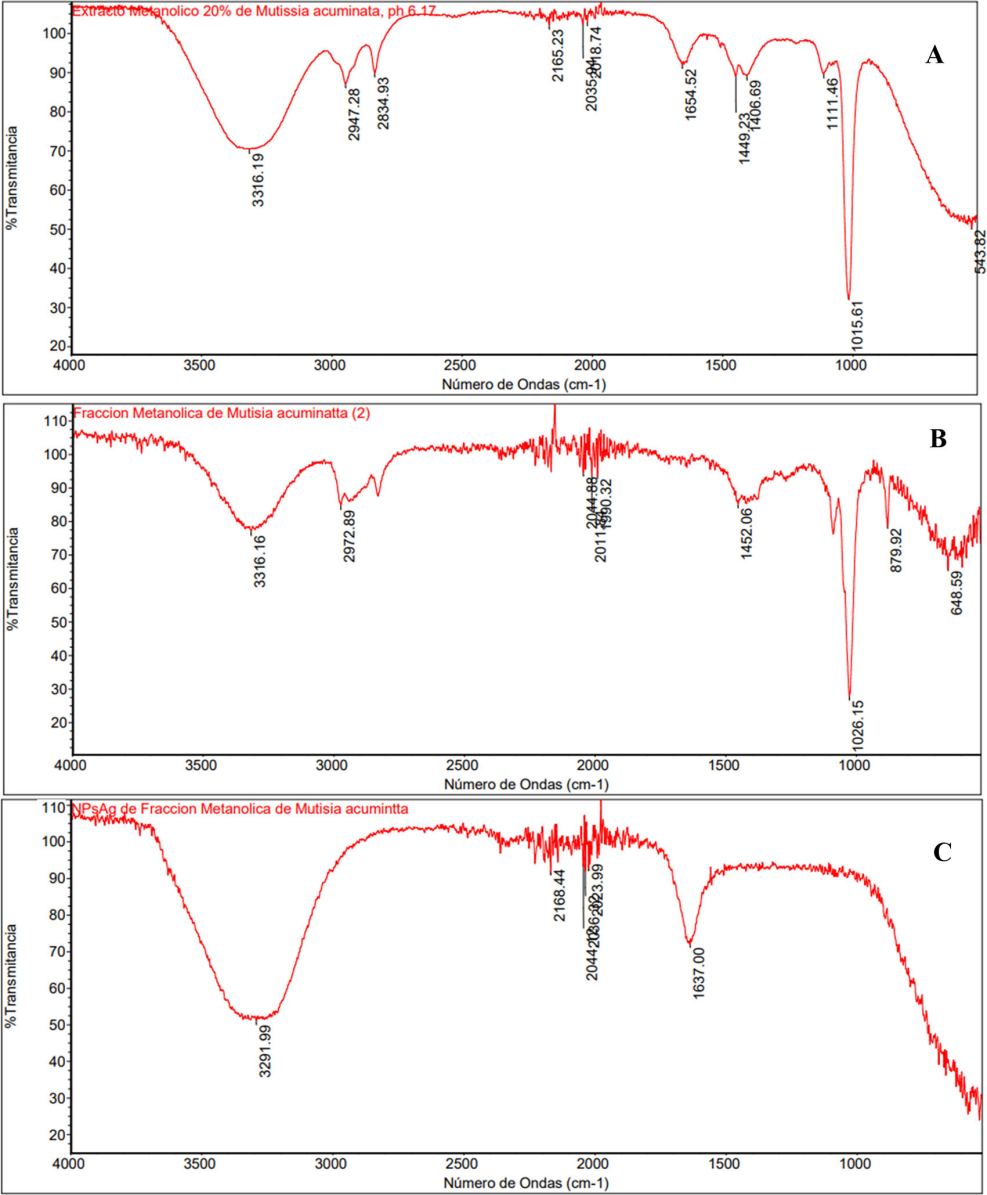
FT-IR spectra. A) Total Methanolic Extract at 20% (EMT); B) Methanolic fraction, C) AgNPs biosynthesized with methanolic fraction.

The FT-IR absorption spectra of AgNPs obtained by green synthesis were studied in the range of 400 cm ^-1^ to 4000 cm ^-1^ wavenumber in transmittance mode. In Figure 4A, 4B and 4C, we can identify a broad band around 3000 cm ^-1^ and 3400 cm ^-1^ approximately due to the N-H stretching vibration of the NH _2_ group and the O-H group of the stretching vibration overlap attributed to the water and phenolic compounds present. Figure 4A, which corresponds to the EMT at 20%, defined transmission peaks were observed at: 3316.19; 2947.28; 2834.93; 1654.52; 1449.23; 1406.69, 1111.46; 1015.61 and 543.82 cm ^-1^. Figure 4B, which corresponds to FM, peaks with lower absorption were observed at: 3616.16; 2972.89; 1452.06; 1026.15; 879.92; 648.59 cm ^-1^, where the disappearance of some IR peaks concerning the total methanolic extract (TME) is appreciated. Figure 4C shows the FTIR spectrum of the biosynthesized AgNPs, bands and spectral peaks of higher intensity and the differences concerning the FTIR spectra of Figure 4A and 4B, where the formation of a peak at 1637.00 cm ^-1^ was observed producing an increase in intensity in the band between 1990.32 and 2500.00 cm ^-1^, which reveals the possible biomolecules of *Mutisia acuminata* extract responsible for reducing silver ions (Ag ^+^) and their interaction with the AgNPs. ^39^ Moreover, notoriously, the absence of several peaks in Figure 4A and 4B corresponding to EMT and FM, respectively, was observed.

### 3.5 Dynamic Light Scattering (DLS)

**Figure 5.**
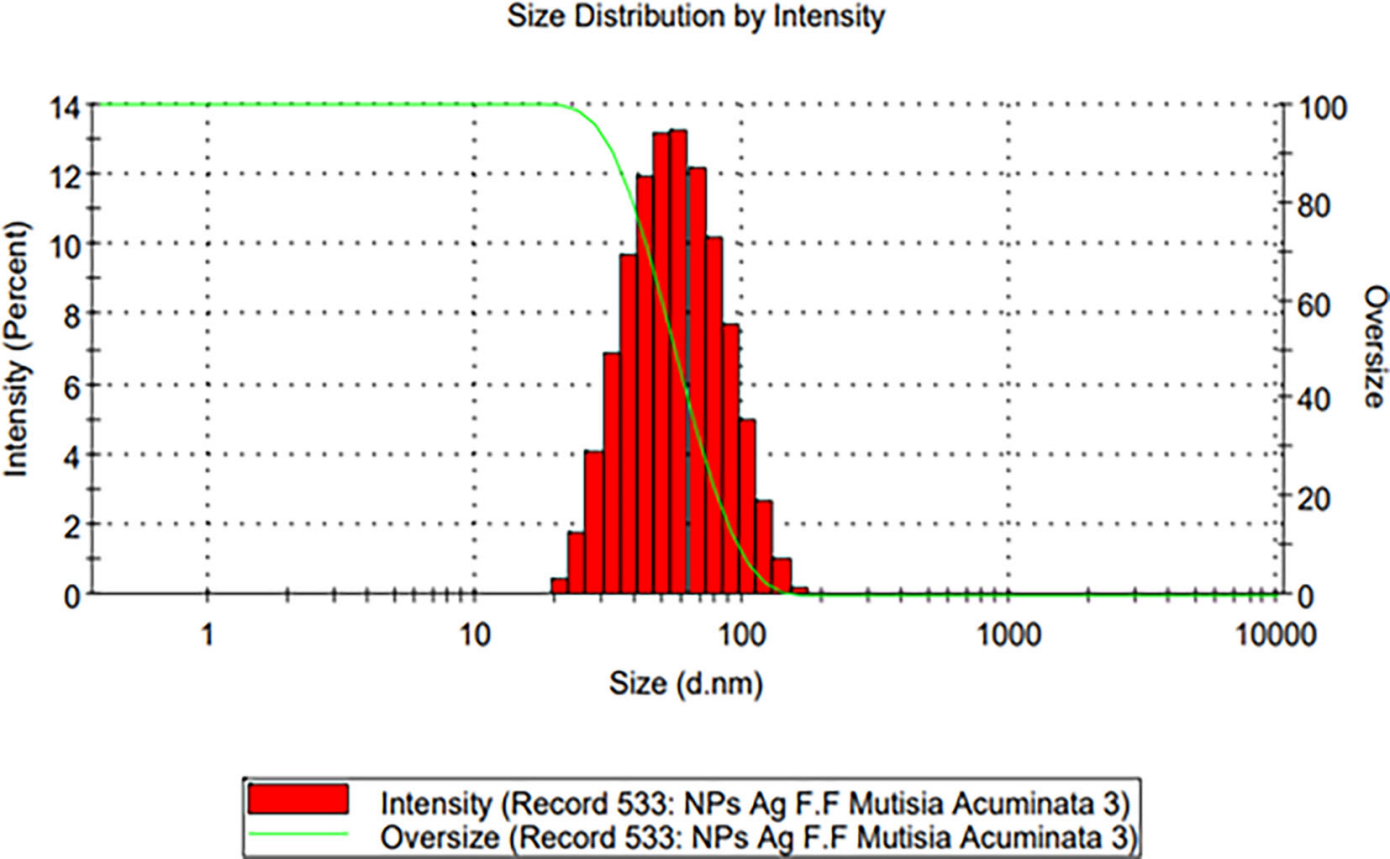
Hydrodynamic size distribution of AgNPs synthesized from the FM of *Mutisia acuminatta*#.

Figure 5 shows the DLS results for the size of the AgNPs obtained in colloidal suspension, which was biosynthesized with the FM of *Mutisia acuminata.* The results show the size distribution of the silver nanoparticles using a monomodal histogram plot with moderate dispersity, presenting hydrodynamic sizes between 20-180 nm approximately and with a polydispersity index (PDI) of 0.260, the average hydrodynamic size (Z- average) was 45.91 nm. The maximum reported size distribution of AgNPs as a function of % intensity was 60.87 nm ± 24.95 nm, as observed in the histogram in Figure 5.

### 3.6 Transmission Electron Microscopy (TEM)

**Figure 6.**
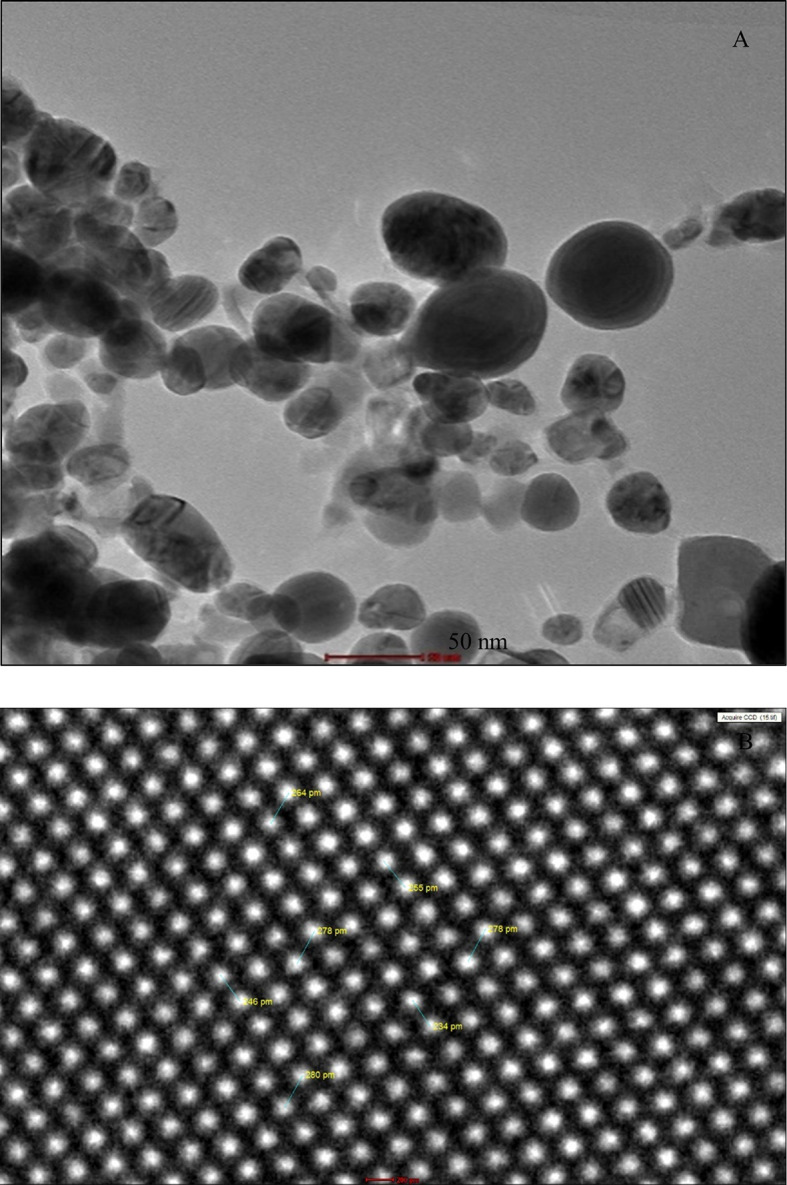
A) TEM micrographs of *Mutisia acuminatta* biosynthesized AgNPs, the nanoscale image of the obtained shape of the AgNPs. B) TEM image of silver with atomic resolution and crystallinity pattern.

Figure 6A shows the results by transmission electron microscopy (TEM), where the biosynthesized AgNPs present a quasi-spherical morphology with agglomerates, the agglomeration observed in Figure 6A could be due to the drying process before analysis by microscopy. ^40^ The size range observed in Figure 6A was between 30 to 60 nm; these results illustrate the formation of AgNPs used as a reducing agent in the phytoconstituents of *Mutisia acuminata* FM. Figure 6A also shows the biomolecular coating on the surface layer of AgNPs, which could be responsible for the stability of the obtained AgNPs. ^39^ Figure 6B, from the TEM micrograph, shows the regular distribution pattern of silver atoms forming the crystal lattice of the obtained nanoparticles, which present a distance between 260-270 pm in the observed interatomic plane. These results could correspond to the interplanar (111) separation of the planes in the cubic crystal structure centered on the faces of the silver atomic arrangement. ^41^

### 3.7 Experimental design and statistical analysis

**3.7.1 Level of influence of the factors according to the Plackett-Burman Design (PBD)**

The Plackett-Burman design (PBD) helps to recognize significant variables with fewer experiments ^38^; the variables were tested at two levels (high and low) indicated by (+1) and (-1), respectively, as shown in Table 1. The PBD design configuration was developed with Minitab 19 software, which predetermined 15 experimental tests of the factorial design, plus 3 central tests, to evaluate the curvature of the design, as presented in Table 2. According to Asfaram *et al*. ^42^ in Table 1, the number of total variables identified for the process of synthesis of AgNPs bi-synthesized Ag with the methanolic fraction (AgNPs-FM) of *Mutissia acuminatta* is summarized, where the independent variables identified by PBD were as follows; i.e., K (number of factors) equal to 6. Likewise, the independent variables (Xi) are also categorized here with their respective denomination and physical units; the upper (+1), lower (-1) and central (0) levels are also presented for each of the variables on the coded scale, and the dependent variable (Y) is also denoted as the response variable. The results (Y) were obtained by spectral scanning using the UV-VIS spectrophotometer, determining the maximum absorbance of the spectrum at a fixed wavelength of 411 nm.

The experimental design was developed to reduce the number of experimental runs. ^38^^,^^42^^,^^43^ As analyzed in Table 1 and Table 2, the concentration effects of stirring speed X _1_ (RPM), potential hydrogen X _2_ (pH), synthesis temperature X _3_ (°C), synthesis time X _4_ (min), the volume of the methanolic fraction X _5_ (μL) and the concentration of AgNO _3_ X _6_ (mM), on the absorbance (411 nm), were investigated empirically using the Plackett-Burman design developed ^44^ in Table 2.

**3.7.2 Analysis of Variance (ANOVA) and significance effect**

**Figure 7.**
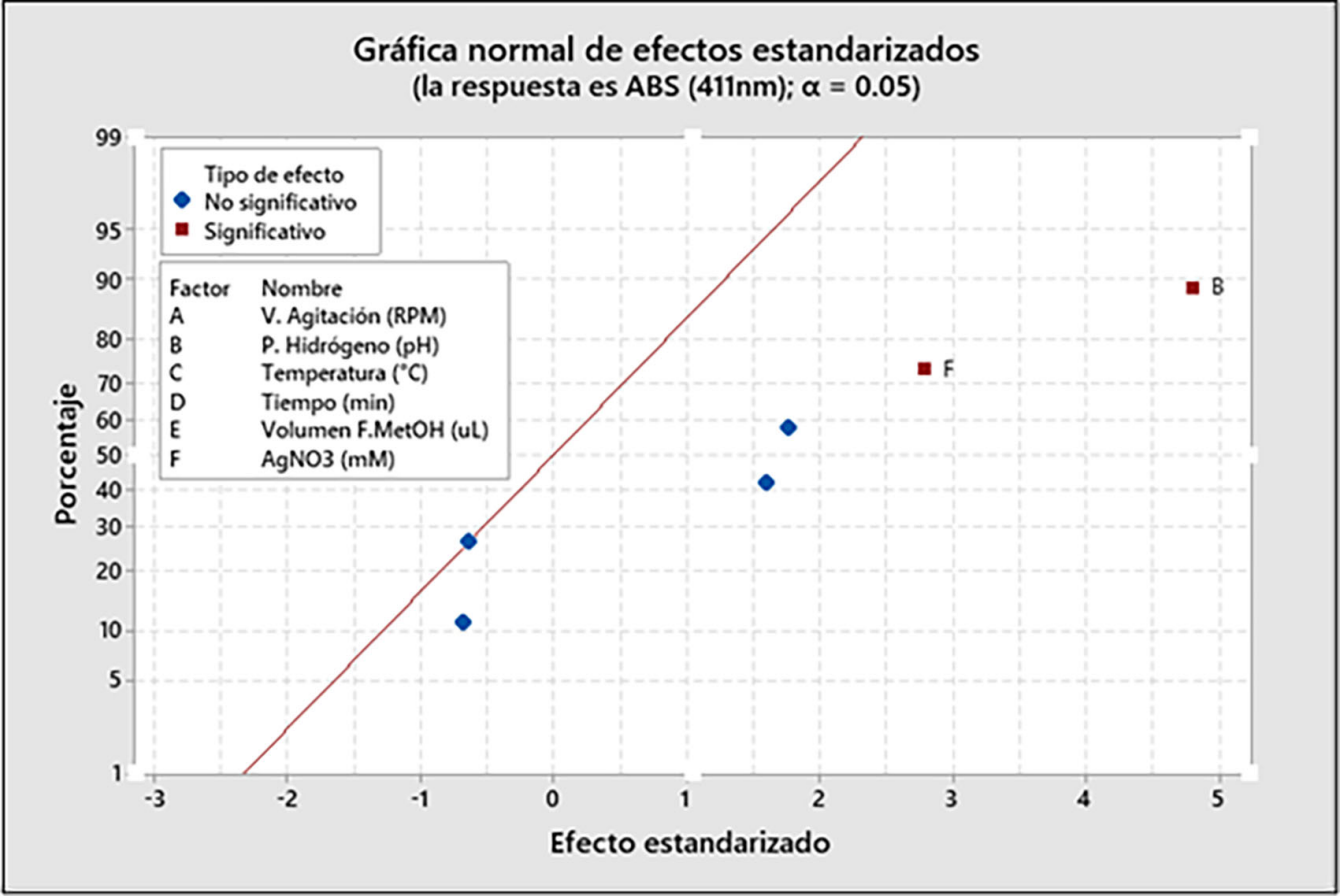
Normal plot of standardized effects.

**Figure 8.**
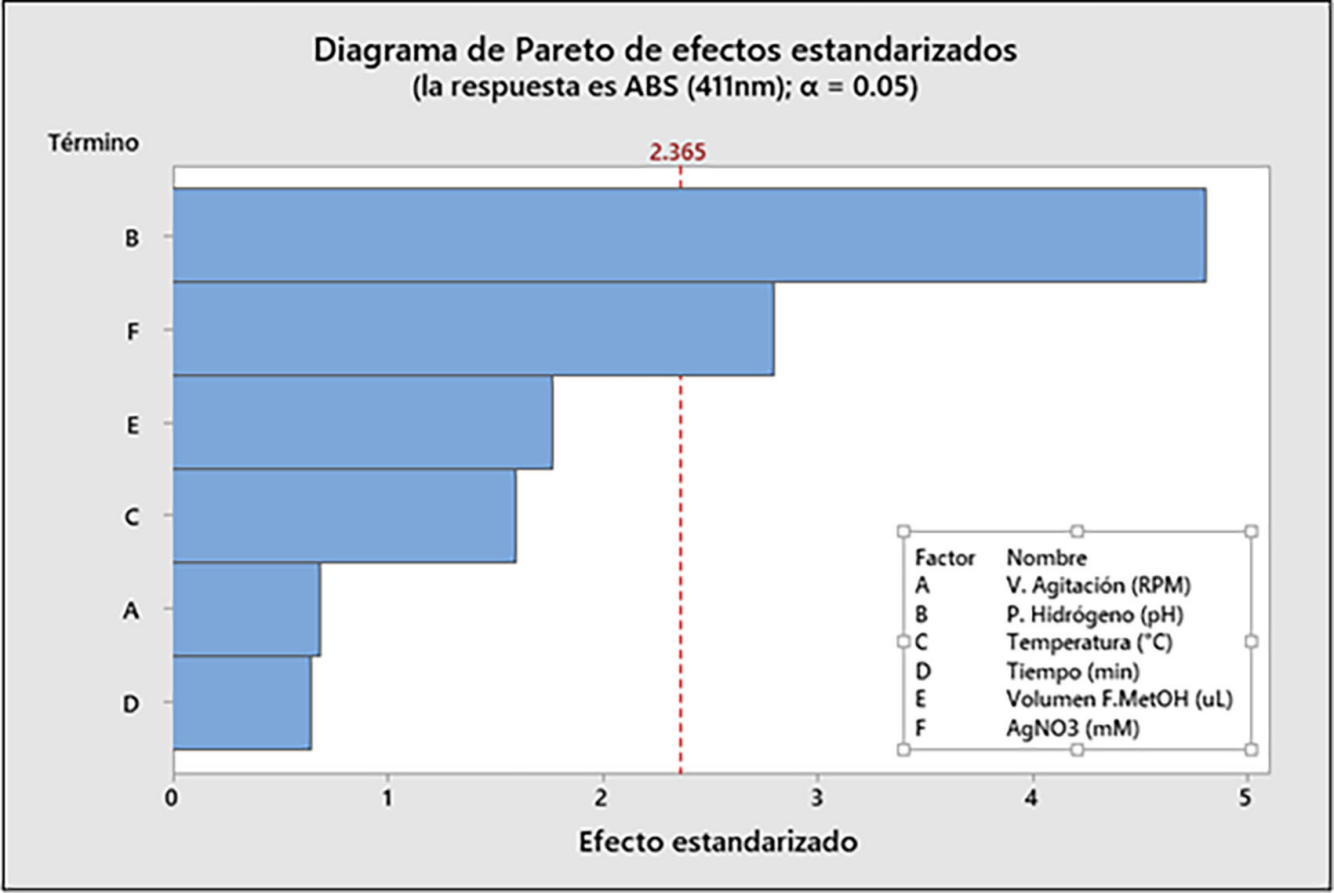
Pareto diagram of standardized effects.

Table 3 presents the ANOVA and significance analysis of the variables; the statistical significance of the model was evaluated and estimated by the F-value (5.40) and the p-value (p < 0.020). The results suggest that the listed factors had remarkable influences on the absorbance (411 nm) recorded, except for the pH of synthesis (X _2_) and AgNO _3_ con-centration (X _6_), due to their low p-value (p-value = ∼0.002 and ∼0.027 < 0.05) respectively. Therefore, these two factors had significance in *Mutisia acuminata* AgNPs biosynthesis, having some impact on the response variable (Y). Therefore, Table 3 shows the results identifying 2 factors that significantly influence the response variable Y (Abs 411 nm). The development of the significance test with the F-statistic and the calculations developed in Table 3 was performed at 95% (p < 0.05), with 1 and 7 degrees of freedom, respectively, for the development of the ANOVA. The standard plot of the effects ( Figure 7) and the Pareto diagram ( Figure 8) are presented, which indicate that of the total of 6 variables evaluated: the hydrogen potential (pH) (X _2_) and the AgNO _3_ concentration (mM) (X _6_) were significant variables of the AgNPs biosynthesis process, with a probability α = 0.05. The results of the Plackett-Burman design of experiments are shown as a standardized Pareto plot in Figure 8. The graphs show the p-values of the independent variables, which were considered to have a significant effect when the values were <0.05, with 95% confidence.

**Table 3.** Analysis of variance (ANOVA) and significance of the variables.

Source	df	SC Sec.	Contribution	SC Ajust.	MC Ajust.	F- value	P- value	Significance
Model	7	2.47245	84.37%	2.47245	0.35321	5.40	0.020	
Lineal	6	2.44840	83.55%	2.44840	0.40807	6.23	0.015	
Agitation Speed (RPM)	1	0.03075	1.05%	0.03075	0.03075	0.47	0.515	No sig.
Hydrogen Potential (pH)	**1**	**1.51109**	**51.56%**	**1.51109**	**1.51109**	**23.09**	**0.002**	**Sig**
Temperature (°C)	1	0.16603	5.67%	0.16603	0.16603	2.54	0.155	No sig.
Time (min)	1	0.02686	0.92%	0.02686	0.02686	0.41	0.542	No sig.
Volume of Methanolic Fraction (uL)	1	0.20324	6.94%	0.20324	0.20324	3.11	0.121	No sig.
AgNO _3_ Concentration (mM)	**1**	**0.51043**	**17.42%**	**0.51043**	**0.51043**	**7.80**	**0.027**	**Sig**
Curvature	1	0.02404	0.82%	0.02404	0.02404	0.37	0.564	
Error	7	0.45816	15.63%	0.45816	0.06545			
Lack of adjustment	5	0.44643	15.23%	0.44643	0.08929	15.23	0.063	
Pure error	2	0.01173	0.40%	0.01173	0.00586			
**TOTAL**	**14**	**2.93060**	**100.00%**					

**3.7.3 Polynomial regression**

The multiple linear regression analysis and the Plackett-Burman factorial design allowed the design of a statistical polynomial model representative of the experimental data. ANOVA determined the significant effect for each variable, and the model’s coefficients were found from this. The significance of the variables evaluated was detected with the T-value and p-values (p < 0.05). The regressions were represented by the corresponding polynomial equations 2 and 3 for the coded and uncoded parameters, respectively:

$$Y=0.5917-0.0506\;\left(X_{1}\right)+0.3549\;\left(X_{2}\right)+0.1176\;\left(X_{3}\right)-0.0473\;\left(X_{4}\right)+0.1301\;\left(X_{5}\right)+0.2062\;\left(X_{6}\right)-0.100\;\mathrm{Pt}\;\text{Ctral}.$$ (2)

$$\mathrm{ABS}\;\left(411\;\mathrm{nm}\right)=-7.66-0.000338\;\left(\mathrm{RPM}\right)+0.710\;\left(\mathrm{pH}\right)+0.0235\;\left({}^{\circ}C\right)-0.00158\;\left(\min\right)+0.00521\;\left(\mathrm{uL}\;F.M\right)+0.412\;\left({\text{AgNO}}_{3}\;\mathrm{mM}\right)-0.100\;\mathrm{Pt}\;\text{Ctral}.$$ (3)

**3.7.4 Adjustment of the polynomial regression model**

**Figure 9.**
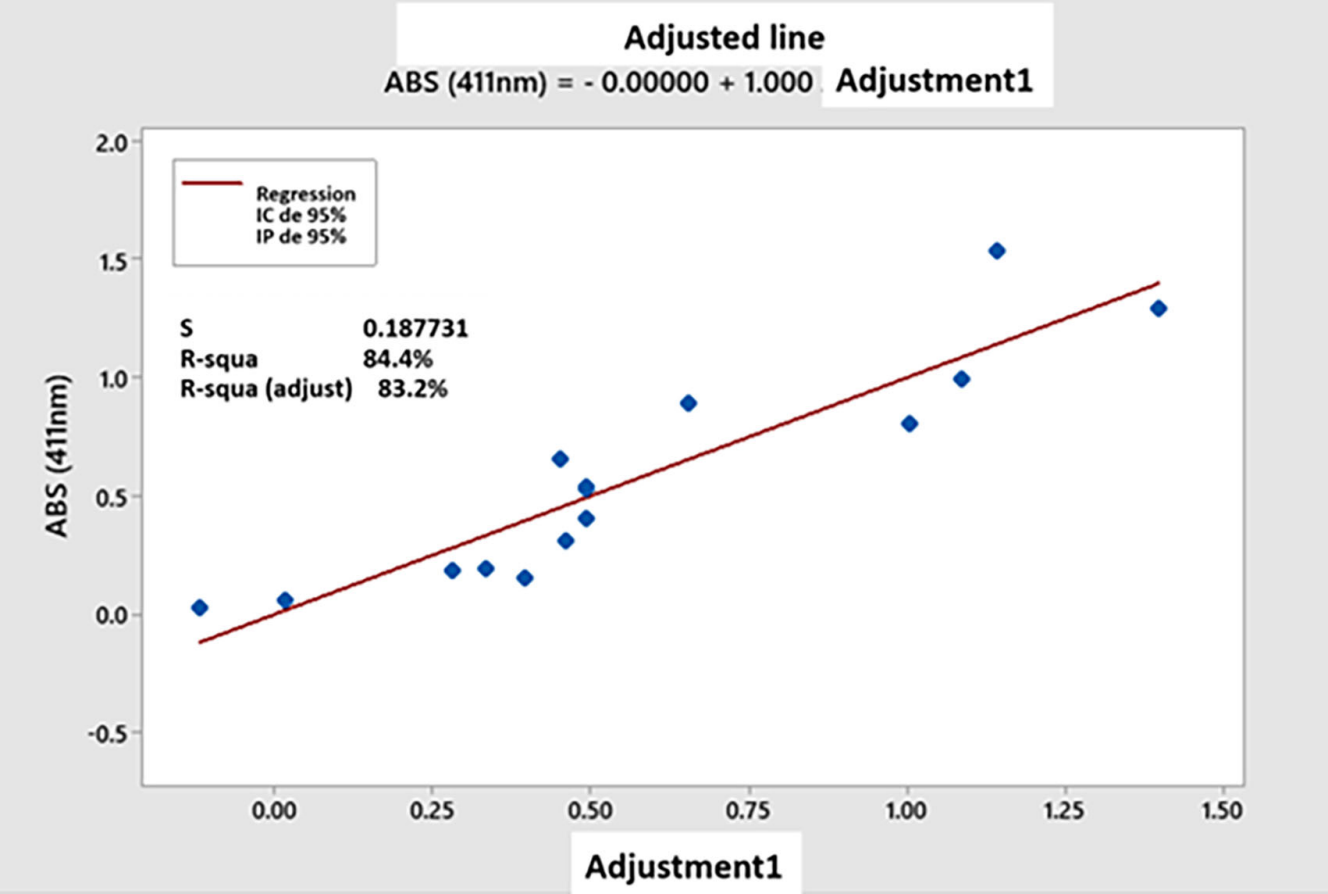
Model fit of experimental data (ABS 41 nm) and estimated data (Fit 1).

Figure 9 represents the fit model of the experimental data and the data estimated from the polynomial regression equation ( equation 2), finding that the value of the correlation coefficient (R ^2^) to be 84.40%, which is a value closely related to the adjusted coefficient of determination (R ^2^ adj.) with a value of 83.20%, showing the acceptability of the empirical data. Therefore, Figure 9 shows the adjusted model of the data obtained, where the scattered points in the graph correlate with fit to the trend line, thus presenting a high adjusted correlation coefficient (Adjusted R ^2^ = 83.20%).

**3.7.5 Contour plots and surface plots of the polynomial model**

**Figure 10.**
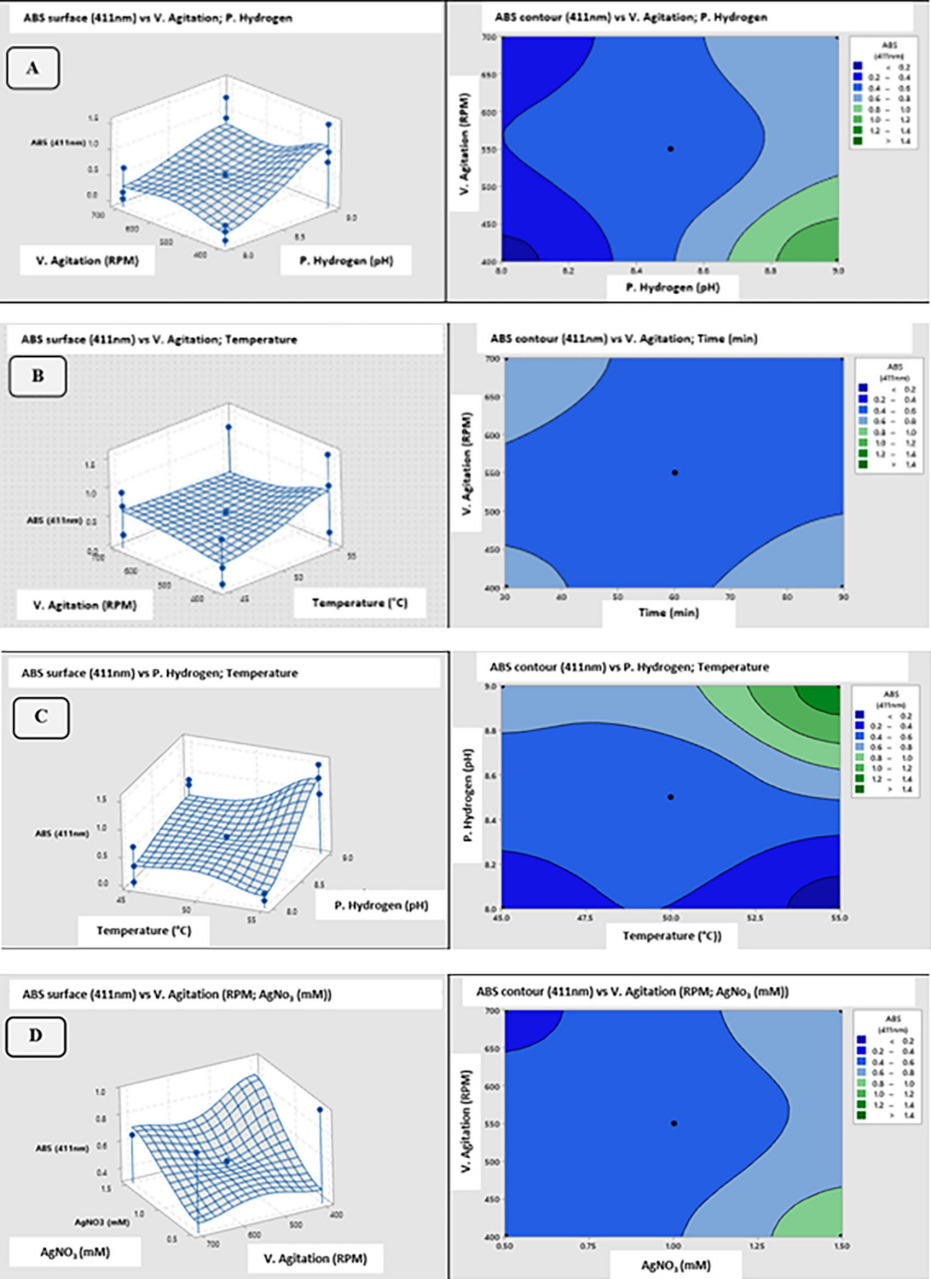
3D response surface and 2D contour plots of the polynomial regression model for ABS (411 nm) as a function of (A) stirring speed (RPM) vs. pH, (B) stirring speed (RPM) vs. temperature, (C) pH vs. temperature, (D) stirring speed (RPM) vs. AgNO _3_.

Figure 10 shows the set of 3D surface plots and 2D contour plots of the obtained polynomial model; these plots show the hyperplanes and contours that were generated by the model for Absorbance (411 nm) as a function of the evaluated variables X _1_ (RPM), X _2_ (pH), X _3_ (°C), X _4_ (min), X _5_ (μL) and X _6_ (mM). Each graph was generated by Minitab 19 statistical analysis software. Surface and contour Figure 10A, B, C and D combine two independent parameters for response prediction (Abs 411nm), as described below: Figure 10A shows the surface and contour plots of Absorbance (411 nm) as a function of stirring speed (RPM) and pH. Figure 10B shows the surface and contour plots of Absorbance (411 nm) as a function of stirring speed (RPM) and temperature (°C). Figure 10C shows Absorbance’s surface and contour plots (411 nm) as a function of pH and temperature (°C). Figure 10D also shows the surface and contour plots of Absorbance (411 nm) as a function of stirring speed (RPM) and AgNO _3_ concentration (mM). Graphs A, B, C and D are response surfaces that present slight curvatures detected by the central points of the PBD. Also, the maximum extremes located for this region of points can be appreciated as effective responses of the polynomial prediction model about the parameters of greater significance than the ANOVA determined.

### 3.8 Antibacterial activity

Table 4 shows the results obtained by the inhibition halos method for the sensitivity analysis of the samples evaluated against the bacterial strains *Escherichia coli* ATCC 33876 and *Staphylococcus aureus* ATCC 25923. According to the data, the most significant antibacterial activity was against *Escherichia coli* ATCC 33876, showing inhibition halos (mm) of greater diameter concerning the *Staphylococcus aureus* ATCC 25923 strain. According to the data in Table 4, the most significant inhibition halos recorded were given by the following samples: (1) AgNPs - FM, (2) EMT (5%), (3) FM and followed by (4) MetOH, highlighting the inhibition effect of biosynthesized silver nanoparticles (AgNPs - FM). According to Table 4, the nanoparticles synthesized with the methanolic fraction (AgNPs - FM) presented higher antibacterial activity against *Escherichia coli* ATCC 33876, with the following average inhibition halos for each cultured plate: 5.167 ± 0.289 mm; 5.333 ± 0.289 mm and 5.167 ± 0.289 mm respectively. However, the NPsAg-FM against *Staphylococcus aureus* ATCC 25923 presented a smaller average inhibition halo: 3.667 ± 0.289 mm; 4.000 ± 1.000 mm; 3.833 ± 0.289 mm.

**Table 4.** Antibacterial effects against *Staphylococcus aureus* ATCC 25923 and *Escherichia coli* ATCC 33876.

Sample	N° Plate	Inhibition Halos (mm)						Average 1	Average 2
		*S. aureus* ATCC 25923			*E. coli* ATCC 33876			S. *aureus* halos (mm)	E. *coli* halos (mm)
EMT 5%	1	2.5	2	3	2	2	2	2.500 ± 0.500	2.000 ± 0.000
	2	3	2	2	2	2	2	2.333 ± 0.577	2.000 ± 0.000
	3	1	2	1.5	2	2	1.5	1.500 ± 0.500	1.833 ± 0.289
FM	4	3.5	2	3	2	1.5	1	2.833 ± 0.764	1.500 ± 0.500
	5	2.5	1.5	1.5	1.5	2	2	1.833 ± 0.577	1.833 ± 0.289
	6	2	1.5	1.5	2	2	1.5	1.667 ± 0.289	1.833 ± 0.289
AgNPs-FM	7	4	3.5	3.5	5	5.5	5	3.667 ± 0.289	5.167 ± 0.289
	8	5	3	4	5.5	5.5	5	4.000 ± 1.000	5.333 ± 0.289
	9	4	3.5	4	5	5.5	5	3.833 ± 0.289	5.167 ± 0.289
MetOH	10	0	1.5	1	2	1.5	2	0.833 ± 0.764	1.833 ± 0.289
	11	1	1	1	1.5	2.5	1.5	1.000 ± 0.000	1.833 ± 0.577
	12	0.5	0.5	0.5	1	2	1.5	0.500 ± 0.000	1.500 ± 0.500

**Figure 11.**
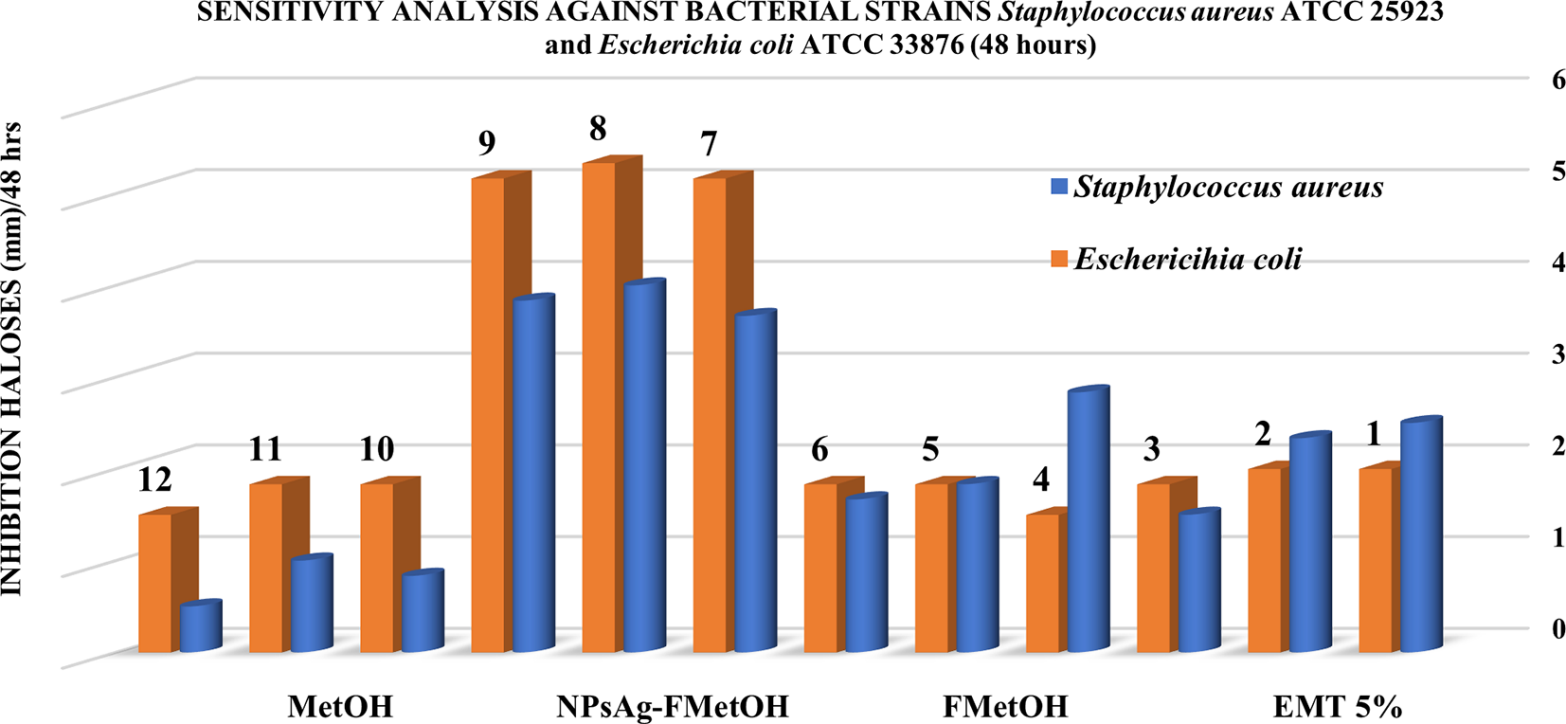
Representation of antibacterial effect against certified strains of *Staphylococcus aureus* ATCC 25923 and *Escherichia coli* ATCC 33876: (a) MetOH, (b) AgNPs - FM, (c) FM and (d) EMT (5%).

On the other hand, Figure 11 shows the categorical graphical representation of the antibacterial effect, where it is clearly distinguished that AgNPs – FM and EMT (5%) show a high sensitivity against both strains *Escherichia coli* ATCC 33876 and *Staphylococcus aureus* ATCC 25923, concerning the other substances evaluated (FM and MetOH).

**Figure 12.**
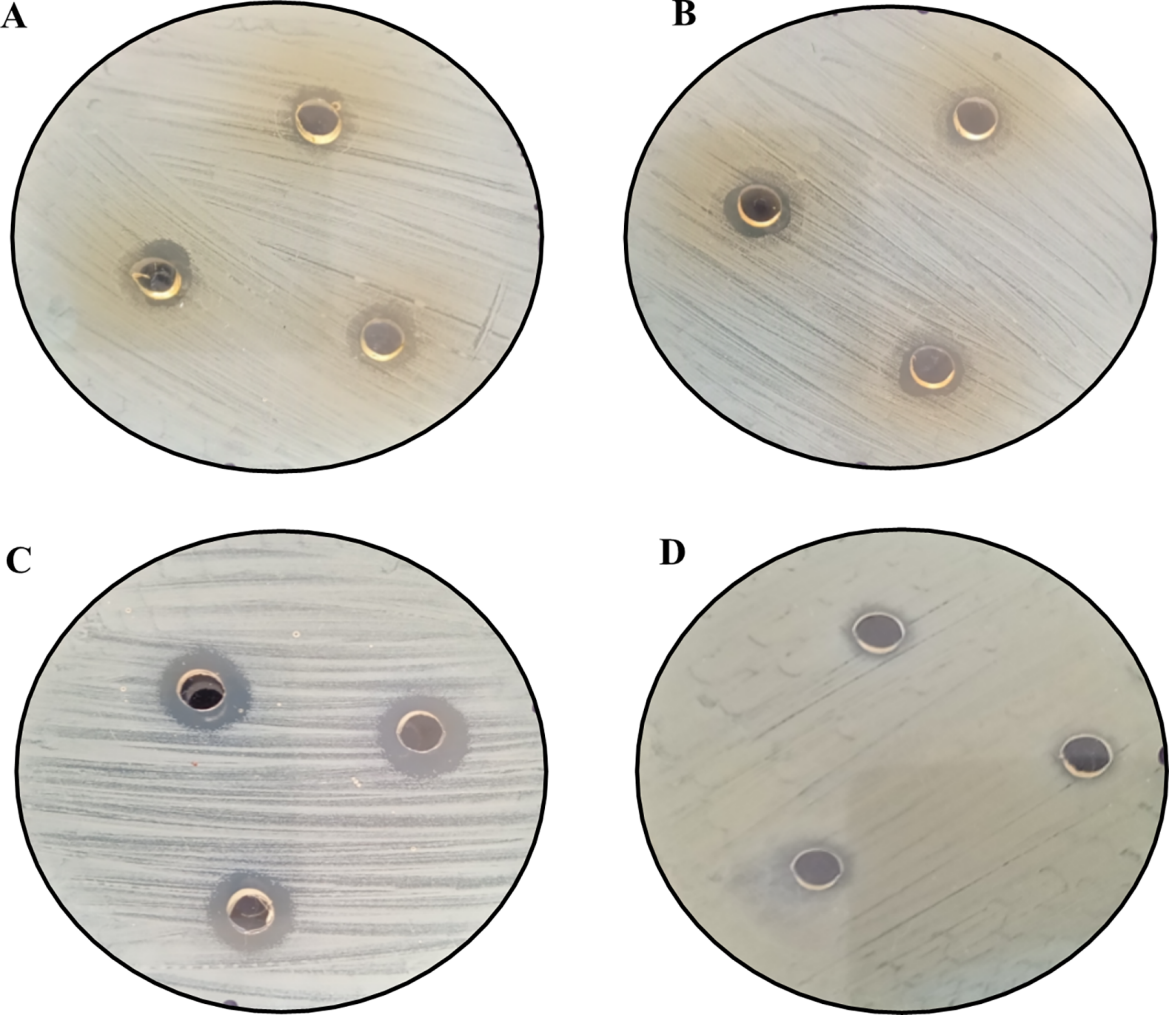
Agar well assays for the evaluation of antibacterial activity against *Staphylococcus aureus* ATCC 25923: (A) EMT (5%), (B) FM, (C) AgNPs-FM and (D) Methanol (blank).

**Figure 13.**
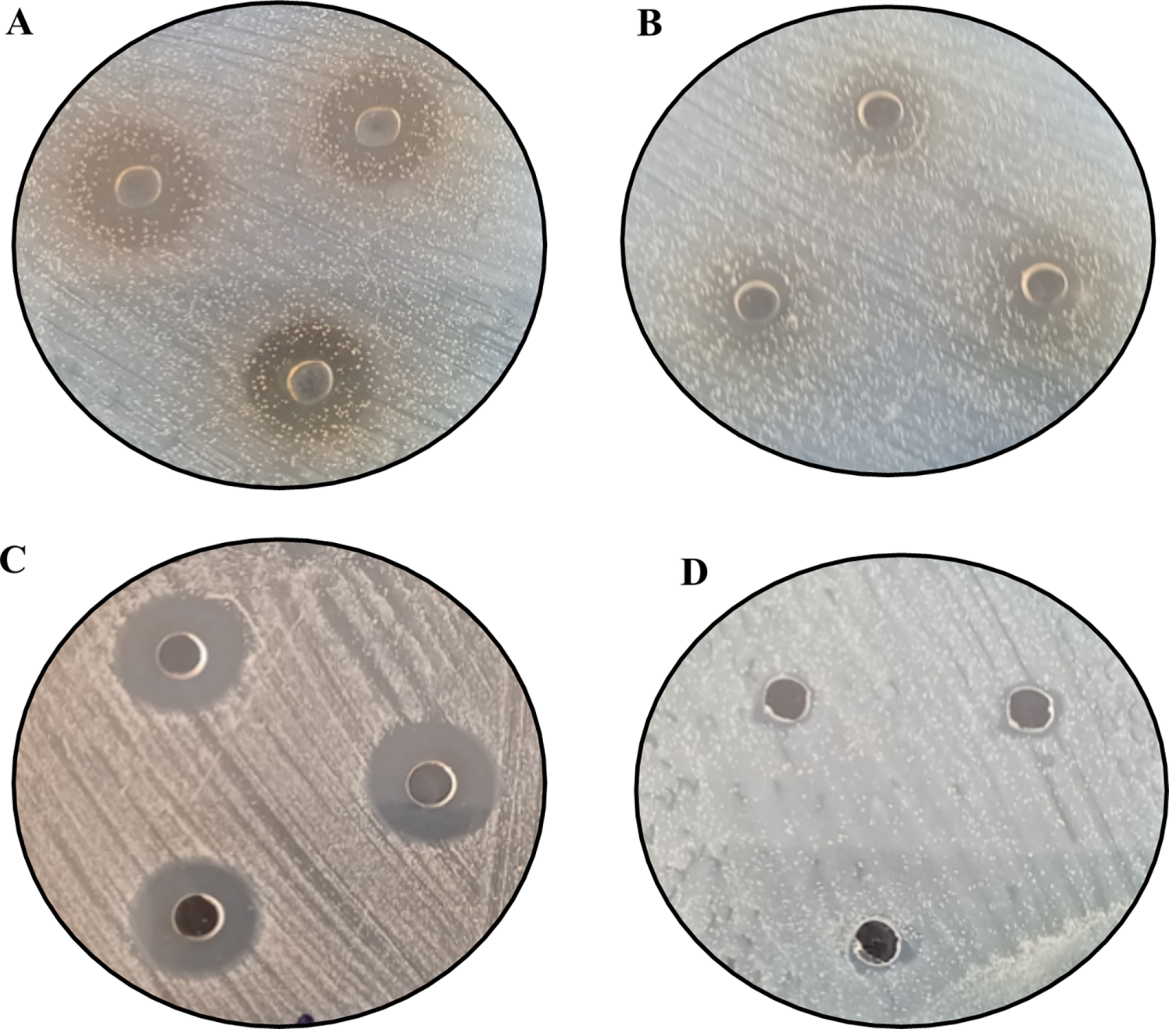
Agar well assays for the evaluation of antibacterial activity against *Escherichia coli* ATCC 33876: (A) EMT (5%), (B) FM, (C) AgNPs-FM and (D) Methanol (blank).

Figures 12 and 13 below show the images of the agar well assay in the Petri dishes, with the respective media and bacteriological strains and the different substances evaluated: (a) EMT (5%), (b) FM, (c) AgNPs-FM and (d) Methanol, against *Escherichia coli* ATCC 33876 and *Staphylococcus aureus* ATCC 25923 respectively, where the formation of the inhibition halos of the medium against the bacterial strains after 48 hours of incubation at 35°C can be observed. The formation of halos of greater average distance for both bacterial strains is given for biosynthesized silver nanoparticles (AgNPs-FM), followed by EMT (5%), presented halos of inhibition with high magnitude of measurement (mm), concerning the other substances as presented in Table 4. The substances that had little sensitivity effect against both bacterial strains were: FM followed by pure methanol with little noticeable inhibition halos and low measurement magnitudes (bacteriostatic), as shown in Table 4.

## 4. Discussion

The preparation of total methanolic extract (TME) of *Mutisia acuminata* leaves has been previously described, ^45^^–^^48^ from which aqueous extracts, hydroalcoholic extracts and purified fractions were consistently obtained. In our work, we obtained a purified methanolic fraction (FM) by column chromatography (LC), TLC ( Figure 1) and HPLC ( Figure 2). Juárez & Mendiondo ^14^ applied these chromatographic separation techniques reporting the presence of flavonoids from the genus *Mutisia acuminata.* On the other hand, the study by Arenas-Chavez *et al*. ^49^ in another plant species called *Lepechinia Meyenii* used column chromatographic (LC) and thin layer chromatographic (TLC) techniques to extract different flavonoid fractions. Laime-Oviedo *et al*. ^2^ used the same qualitative techniques by LC and TLC chromatography to identify the flavonoids of *Lepechinia meyenii* using a chromatographic profile by UHPLC-MS identifying different bioactive compound that are involved as reductants in the biosynthesis of AgNPs. In our study we identified the following secondary metabolites: caffeic acid, coumarins, flavonoids, lignans and nitrogenous derivatives of coumarin, which had participation in the reduction of silver ions from the precursor salt (Ag+) to AgNPs (Ag0). On the other hand, the work developed by Juárez & Mendiondo ^14^ reported the presence of the following metabolites: quercetin, quercetin-3-glucuronide, isorhamnetin-3-glucuronide and pelargonidin diglycoside, which are flavonoids related to the metabolites and flavonoids identified in our plant species of *Mutisia acuminata.*

By UV-VIS spectrophotometry, a maximum absorption peak was observed around 415-420 nm approximately, corresponding to the surface plasmon resonance (SPR) of the AgNPs formed; this maximum absorption peak in the UV-VIS spectrum confirms the formation of the AgNPs, through the biosynthesis carried out with the FM of *Mutisia acuminata.* By DLS, an average hydrodynamic size of 43.71 nm was found for the obtained silver nanoparticles, with a size distribution of monomodal type, according to the data in Figure 5. These results are in agreement with Anarjan *et al*. ^50^ and Eskandari *et al*. ^51^ that reported a similar hydrodynamic size of the nanoparticles. However, they suggested that the particle size distribution was monomodal compared to the polymodal distribution we obtained. It is important to note that the distribution of polymodal particles can accelerate the Oswald ripening of the particles and decrease the physical stability of the NPs systems. Furthermore, the presence of extra hydrate layers, together with ions or molecules attached to the nanoparticle surface in an aqueous environment could be responsible for the larger hydrodynamic sizes. These attached molecules may consist of phytoconstituents such as coumarins, flavonoids and lignans derived from the plant species *Mutisisa acuminata.* An additional contributor, to some extent, could be aggregation as reported in other studies. ^52^^–^^54^ On the other hand, Seabra *et al*. ^40^ reported similar DLS results for biogenically synthesized AgNPs by catechin, the primary polyphenol present in green tea extract with an average hydrodynamic size of 44 nm. The hydrodynamic size of the nanoparticles was found to be more extensive compared to the average size of nanoparticles analyzed by TEM, attributed to extra hydrate layers in aqueous environments. ^53^ The results indicate the formation of nanometer-scale AgNPs in aqueous suspension with the phytoconstituents of *Mutisisa acuminata* FM, especially coumarins that were signals detected by UHPLC-MS, as observed in the chromatogram presented by Figure 2C and the PDI value indicates that the size distribution is moderate polydisperse. ^40^

The FTIR spectra of the methanolic extract of *Mutisia acuminata* (EMT 20%) are shown in Figure 6A; as observed in the spectrum, several of the transmission peaks were centered at: 3316.19; 2947.28; 2834.93; 1654.52; 1449.23; 1406.69, 1111.46; 1015.61 and 543.82 cm ^-1^. The broadest IR spectrum of the absorption peak of the extract was observed at: 3316.19 cm ^-1^; it may refer to the vibration of primary amine stretching and hydroxyl (-OH) groups, which are related to alcohols, flavonoids, phenolic acids. ^27^^,^^55^^–^^57^ The band between 2947.28 to 2834.93 cm ^-1^ indicates the presence of carboxylic acids, and the band at 1654.52 is another representative range for carboxylic acids (1610-1550 cm ^-1^). ^58^ The peak centered at 1015.61 cm ^-1^ can be attributed to the -CO stretching vibrations of carboxylic acid, ester and ether groups of phytoconstituents present in the extract. ^39^^,^^51^^,^^59^^,^^60^ These results are also in agreement with the study carried out by Ghazal *et al*., ^61^ which considered that the appearance and change of position of the peaks at 3600 to 3200 cm ^-1^, 1610 to 1550 cm ^-1^ indicates the presence of OH groups and carboxylic acids respectively and probably NH amine groups, in the protection agents acting as stabilizing agents of the synthesized NPs. The peak at 1111.46 cm ^-1^ could be consigned to the C-N stretching vibration for aromatic and aliphatic amines. ^55^^,^^62^ The corresponding peaks between 1654.52 and 1406.69 cm ^-1^, in the IR spectra for both the extract and AgNPs, respectively, could be attributed to the presence of amide I and amide II arising due to carbonyl stretching vibration and NH stretching. ^63^ Therefore, these results correlate to the data obtained by Salguero & Pilaquinga, ^64^ which distinguish a peak around 1648.02 cm ^-1^ showing a medium intensity band, which belongs to the N-H tension bond corresponding to primary amides, whose deformation and bandwidth generated from the stretching of the carbonyl group in amino acids.

In Figure 6B, which is attributed to the FTIR spectra of the FM, peaks with lower absorption are observed, revealing at: 3616.16; 2972.89; 1452.06; 1026.15; 879.92; 648.59 cm ^-1^, with the disappearance of some IR peaks concerning the extract. The vibrational stretching band at 3616.16 cm ^-1^ corresponds to the -OH of methanol (constituent solvent of FM); in the same way, this vibrational band is presented in the IR spectrum of the extract but with lower absorption. According to Jyoti *et al*., ^65^ the band in the range of 3000 to 3400 cm ^-1^ is the indicator of the stretching of the (OH-) group within groups of the free hydroxyls or may be an indicator of OH- groups attached to aromatic structures, which confirms the existence of phenolic compounds in FM. According to the results obtained by Escobar Falconi, ^66^ the band is located at 2972.89 cm ^-1^; this is formed due to the stretching of the -CH _2_ bond. We can also observe an intense sharp peak shifted to 1026.15 cm ^-1^, compared to the peak in Figure 6A, which is attributed to the stretching vibrations of the carboxylic acid (-C-O-), ester and ether groups. ^39^ These recorded peaks are in agreement with the study performed by Camacho Polo & Deschamps Mercado, ^67^ who distinguish absorption bands at 1043.63 and 1086.15 cm ^-1^ that is related to C-OH, H _2_O and -OH groups. The sharp peaks around 2972.89 cm ^-1^ can be attributed to -OH and C=O stretching vibrations, indicating the presence of aromatic, carbonyl groups and metabolites present in the *Mutisia acuminata* leaf extract, which may be involved in the reduction process. ^39^^,^^68^ Figure 6B reveals a FTIR profile, similar to Figure 6A, the band at 3616.16 cm ^-1^ is associated with stretching solid vibrations of the hydroxyl (-OH) group in the system, ^30^^,^^39^^,^^69^^–^^71^ assigned to the single bond polyol group and single bond vibration H and CH _2_ of phenolic compounds. ^52^^,^^69^ The band of low absorbance peaks corresponding to wavenumber 1452.06 cm ^-1^ ( Figure 6B) is related to stretching carbonyl groups, NH and NH _2_^51^ and -O-H bonds of carboxylic acids. The FTIR spectra of the synthesized AgNPs are shown in Figure 6C, which reveals the possible biomolecules present in the *Mutisia acuminata* extract responsible for the reduction of silver ions (Ag+) and their interaction with the AgNPs. ^39^ The AgNPs spectra show intense bands, and spectral differences are observed concerning the FTIR spectra in Figure 6A and 6B, the formation and position of a new peak at 1637.00 cm ^-1^ and producing an increase in intensity in the band between 1990.32 and 2500.00 cm ^-1^, occurring due to the contribution to the reduction and stabilization process. ^27^ An intense sharp peak is also presented at 1026.15 cm ^-1^, being found slightly shifted about the IR spectrum in Figure 6A, which is attributed to the vibrations due to stretching of the -C-O- carboxylic group. Most notably, several characteristic peaks are absent in the FTIR spectra in Figure 6A and 6B, corresponding to the extract and FM, respectively. Transmission electron microscopy (TEM) identified spherical-shaped NPs with sizes between 20-60 nm in diameter.

The effect of the parameters about the maximum absorbance was given by the Plackett-Burman design (PBD), being analyzed statistically using an ANOVA, from which a polynomial statistical model representative of the experimental data was obtained. Therefore, by running different operators and parameters simultaneously, the most significant variables of the process were determined. On the other hand, it was possible to detect the curvature of the estimated model with the central points of the PBD, while the replicates of the central point provided us with the model’s curvature and a numerical value of the error. ANOVA and significance analysis indicated the most significant effects of the variables for the best absorbance at a fixed wavelength of 411 nm. The methanolic fraction (FM) of *Mutisia acuminata* was used as a bioreductive agent in the synthesis of AgNPs and has been considered as the primary variable, as well as pH (X _2_) and AgNO _3_ precursor salt concentration (X3), which played an essential role in the reaction synthesis and stability of the AgNPs. However, ANOVA evaluated the statistical significance of the model, estimated by F-value (5.40) and p-value (p < 0.020), where all factors had remarkable influences on the absorbance (411 nm) recorded, except synthesis pH (X _2_) and AgNO _3_ concentration (X _6_), due to their low p-value (p-value = ∼0.002 and ∼0.027 < 0.05) respectively. Therefore, these two factors have greater significance in the biosynthesis of AgNPs, having some impact on the response variable Y (Absorbance 411nm), according to Table 4. The development of the significance test with the F-statistic and the calculations developed in Table 4 was performed at 95% (p < 0.05), with significance levels of 1 and 7 degrees of freedom, respectively. Therefore, the results suggest that, among the six factors studied about the response variable (Abs 411 nm), the reaction pH (X _2_) and the concentration of the AgNO _3_ precursor salt used (X _6_) were the most significant variables for the maximum absorbance results (411 nm).

Equation 2, obtained by polynomial regression, was represented by the following coded statistical model: Y = 0.5917-0.0506 (X _1_) + 0.3549 (X _2_) + 0.1176 (X _3_) − 0.0473 (X _4_) + 0.1301 (X _5_) + 0.2062 (X _6_) - 0.100 P.Ctral; which is representative of the experimental data for linear predicted values. However, using 3D response surface and 2D contour plots, the interaction of the most significant variables of AgNPs biosynthesis could be graphically analyzed as a function of absorbance (411 nm), and the curvature of the evaluated design could be detected. To check how the model has fit the prediction, the coefficient of determination (R2) value, which was closer to 100, implied the best prediction of the correlation between experimental and predicted responses. ^33^ Meanwhile, the fit of our polynomial regression model indicated an adequate adjusted correlation coefficient (R ^2^) between the predicted (estimated) response values and the experimental (observed) response values for Absorbance (411nm), indicating to us that the model presented a high correlation, demonstrating the level of accuracy of the prediction. Therefore, it is important to note that the R ^2^ value of a good model is within a range close to 100. The fit of the values was good, and the correlation coefficient was R ^2^ adj = 83.2%, so the polynomial model obtained by the PBD adequately represented the experimental data of the process.

The silver nanoparticles biosynthesized with the methanolic fraction (AgNPs-FM) showed enhanced antibacterial activity against certified bacteriological strains of *Staphylococcus aureus* ATCC 25923 and *Escherichia coli* ATCC 33876. The formation of halos with greater sensitivity against both strains is given by the biosynthesized silver nanoparticles (AgNPs-FM) and EMT (5%), presenting inhibition halos of greater distance; that is, with high measurement magnitudes (mm), concerning the other substances, as can be seen in Table 4 and Figure 12 and 13. According to Ortiz Aguilar, ^72^ this may be because the composition of the cell wall of *Escherichia coli* (gram negative) has a thin layer of peptidoglycan and lipopolysaccharide, which allows a more significant interaction of the nanoparticles with the outer membrane, causing inhibition of active transport and as a consequence inhibition of RNA, DNA and protein synthesis. On the other hand, Nikaeen *et al*. ^61^ reported that the antimicrobial effects found for nanoparticles can be partially explained due to the binding capacity of nanoparticles to the bacterial cell membrane, which can lead to an increase in membrane permeability, also means that nanoparticles can alter the enzymatic activity of bacteria, through the interaction with sulfhydryl (SH) groups of bacterial enzymes.

## 5. Conclusion

The synthesized AgNPs offer a viable option for further development due to the presence of bioactive compounds, adequate characterization and antibacterial activity.

## Data Availability

Figshare: Data - Plackett - Burman design for the detection of the most significant parameters in the biosynthesis of silver nanoparticles with Mutisia acuminatta and evaluation of their antibacterial effect, https://doi.org/10.6084/m9.figshare.23897037.v1. ^73^ This project contains the following underlying data:
• Absorbance for the methanolic fraction • Absorbance for the synthesis kinetics • FT-IR spectra Data are available under the terms of the Creative Commons Attribution 4.0 International license (CC-BY 4.0).
