# Supplementary material for: Plackett-Burman design in the biosynthesis of silver nanoparticles with Mutisia acuminatta (Chinchircoma) and preliminary evaluation of its antibacterial activity
Source: F1000Res. 2023 Nov 13;12:1462. [Version 1] doi: 10.12688/f1000research.140883.1 (PMC10905015; doi:10.12688/f1000research.140883.1)
Supplement: Peer review history

# Reviewer report: Reviewer response for version 1

**DOI:** 10.5256/f1000research.154287.r232484

**Published:** 2024-02-09

**Authors:** Davoodbasha Mubarakali

**Affiliations:** 1 School of Life Sciences, B.S. Abdur Rahman Crescent Institute of Science and Technology, Chennai, Tamil Nadu, India

The manuscript requires severe revision for consideration for indexing. However, the article emphasized the optimization of silver nanoparticle synthesis using PBD, so the whole manuscript needs to be revised. The following comments might be helpful:

- Fig. 3, y axis unit.
- Fig. 4, x and y axis are not in English.
- Fig. 5 - Caption. 
- The author have to make sure of the name of the plant material, Mutisia acuminatta or Mutisia acuminata.
- Fig. 7, x and y axis are not in English.
- Fig. 8, x and y axis are not in English.
- Insufficient statement: 5. Conclusion The synthesized AgNPs offer a viable option for further development due to the presence of bioactive compounds, adequate characterization and antibacterial activity

Is the work clearly and accurately presented and does it cite the current literature?

Partly

If applicable, is the statistical analysis and its interpretation appropriate?

Partly

Are all the source data underlying the results available to ensure full reproducibility?

Partly

Is the study design appropriate and is the work technically sound?

Yes

Are the conclusions drawn adequately supported by the results?

No

Are sufficient details of methods and analysis provided to allow replication by others?

Partly

Reviewer Expertise:

NA

I confirm that I have read this submission and believe that I have an appropriate level of expertise to confirm that it is of an acceptable scientific standard, however I have significant reservations, as outlined above.

# Reviewer report: Reviewer response for version 1

**DOI:** 10.5256/f1000research.154287.r222787

**Published:** 2024-02-09

**Authors:** Amr Mohamed Abdelghany

**Affiliations:** 1 Spectroscopy Department, Physics Research Institute, National Research Centre, Dokki, Giza, Egypt

Based on my review, here are some suggestions to enhance the manuscript:

**Introduction:**

- Provide more background on the plant Mutisia acuminata and its potential for nanoparticle synthesis. Elaborate on any prior studies using this plant.
- Expand on the motivation for using a green synthesis approach with plant extracts. Highlight the advantages over chemical synthesis.
- Introduce the Plackett-Burman experimental design and explain why it was selected for this study.

**Methods :**

- Give more details on the plant extract preparation - solvents used, extraction technique, temperature, time etc. This will allow reproducibility.
- Provide information on the concentrations/volumes of plant extract and silver nitrate used in the synthesis.
- Include synthesis parameters such as temperature, pH, time for the nanoparticle formation reaction.
- Specify the instruments used for UV-vis, FTIR, DLS characterization along with operating conditions.

**Results/Discussion:**

- Include TEM and SEM micrographs to show nanoparticle morphology. Particle size distribution analysis would also strengthen the characterization.
- Provide FTIR spectra of plant extract alongside nanoparticles to identify shifts in functional groups due to nanoparticle binding.
- Elucidate the possible phytochemicals responsible for reduction and stabilization of nanoparticles based on FTIR data.
- Discuss proposed mechanisms for enhanced antibacterial activity - nanoparticle cell wall interactions, ROS generation etc.
- Perform cytotoxicity studies on mammalian cells to demonstrate biocompatibility.
- Evaluate the nanoparticles for additional applications like catalysis, drug delivery etc based on the green synthesis approach.

**Conclusions:**

- Summarize the key outcomes - nanoparticle synthesis, characterization and antibacterial activity.
- Highlight the advantages of the green synthesis method using Plackett-Burman optimization.

Overall, providing more experimental details, expanding the nanoparticle characterization and applications, and discussing the synthesis mechanisms would significantly improve the manuscript. The results demonstrate a thorough study, but the manuscript would benefit from more data on NP characterization and antibacterial activity.

Is the work clearly and accurately presented and does it cite the current literature?

Yes

If applicable, is the statistical analysis and its interpretation appropriate?

I cannot comment. A qualified statistician is required.

Are all the source data underlying the results available to ensure full reproducibility?

Yes

Is the study design appropriate and is the work technically sound?

Yes

Are the conclusions drawn adequately supported by the results?

Yes

Are sufficient details of methods and analysis provided to allow replication by others?

No

Reviewer Expertise:

Material science and spectroscopy

I confirm that I have read this submission and believe that I have an appropriate level of expertise to confirm that it is of an acceptable scientific standard, however I have significant reservations, as outlined above.

# Reviewer report: Reviewer response for version 1

**DOI:** 10.5256/f1000research.154287.r232483

**Published:** 2024-01-03

**Authors:** Samson O Aisida

**Affiliations:** 1 Department of Physics and Astronomy, University of Nigeria, Nsukka, Enugu, Nigeria

The authors presented

 Plackett-Burman design in the biosynthesis of silver nanoparticles with *Mutisia acuminatta* (Chinchircoma) and preliminary evaluation of its antibacterial activity

 Few comments are listed below before acceptance.

 Minor review

 Introduction line 18 and 19

1. The presence of the various peaks determines the possible bioactive compounds that can reduce Ag+ (the plus should be superscript) ions
2. Section 2.4.4, line 1

The biosynthesized NPsAg AgNPs

1. Section 3.2, line 10

complex formed between silver and the solvent AgCH3CN+ at t = 1.95 min. Check this equation

1. figure 1 is fussy, produce better one
2. figure labelling unit should be corrected
3. the figures label can be corrected to English
4. the conclusion should be populated.
5. The introduction should be updated with the following current articles

https://doi.org/10.1016/j.matchemphys.2019.121859

https://doi.org/10.1016/j.matchemphys.2019.121859

https://doi.org/10.1016/j.surfin.2019.100359

https://doi.org/10.1007/s10965-019-1897-z

https://doi.org/10.1016/j.surfin.2019.100419

https://doi.org/10.1016/j.matpr.2020.03.005

https://doi.org/10.1016/j.matpr.2020.02.931

https://doi.org/10.1016/j.mtcomm.2022.104660

Is the work clearly and accurately presented and does it cite the current literature?

Yes

If applicable, is the statistical analysis and its interpretation appropriate?

Yes

Are all the source data underlying the results available to ensure full reproducibility?

Yes

Is the study design appropriate and is the work technically sound?

Yes

Are the conclusions drawn adequately supported by the results?

Yes

Are sufficient details of methods and analysis provided to allow replication by others?

Yes

Reviewer Expertise:

Nanomaterials

I confirm that I have read this submission and believe that I have an appropriate level of expertise to confirm that it is of an acceptable scientific standard.

## References

[1] : Biogenic synthesis and antibacterial activity of controlled silver nanoparticles using an extract of Gongronema Latifolium. *Materials Chemistry and Physics* .2019;237: 10.1016/j.matchemphys.2019.121859 10.1016/j.matchemphys.2019.121859

[2] : Biosynthesis of silver nanoparticles using bitter leave (Veronica amygdalina) for antibacterial activities. *Surfaces and Interfaces* .2019;17: 10.1016/j.surfin.2019.100359 10.1016/j.surfin.2019.100359

[3] : Incubation period induced biogenic synthesis of PEG enhanced Moringa oleifera silver nanocapsules and its antibacterial activity. *Journal of Polymer Research* .2019;26(9) : 10.1007/s10965-019-1897-z 10.1007/s10965-019-1897-z

[4] : Concentration induced properties of silver nanoparticles and their antibacterial study. *Surfaces and Interfaces* .2020;18: 10.1016/j.surfin.2019.100419 10.1016/j.surfin.2019.100419

[5] : Biosynthesis of silver oxide nanoparticles using leave extract of Telfairia Occidentalis and its antibacterial activity. *Materials Today: Proceedings* .2021;36: 10.1016/j.matpr.2020.03.005 208-213 10.1016/j.matpr.2020.03.005

[6] : Morphological, optical and antibacterial study of green synthesized silver nanoparticles via Vernonia amygdalina. *Materials Today: Proceedings* .2021;36: 10.1016/j.matpr.2020.02.931 199-203 10.1016/j.matpr.2020.02.931

[7] : Biogenic synthesis of zinc oxide nanorods for biomedical applications and photodegradation of Rhodamine B. *Materials Today Communications* .2022;33: 10.1016/j.mtcomm.2022.104660 10.1016/j.mtcomm.2022.104660
